# Diagnosis of Perinatal Mental Health Conditions Following Medicaid Expansion to Include Low-Income Immigrants

**DOI:** 10.1001/jamanetworkopen.2024.0062

**Published:** 2024-02-20

**Authors:** Maria I. Rodriguez, Ann Martinez-Acevedo, Menolly Kaufman, Erin C. Nacev, Kristen Mackiewicz-Seghete, K. John McConnell

**Affiliations:** 1 Department of Obstetrics and Gynecology, Oregon Health & Science University, Portland; 2 Center for Health Systems Effectiveness, Oregon Health & Science University, Portland; 3 Department of Psychiatry, Oregon Health & Science University, Portland

## Abstract

**Importance:**

For some low-income people, access to care during pregnancy is not guaranteed through Medicaid, based on their immigration status. While states have the option to extend Emergency Medicaid coverage for prenatal and postpartum care, many states have not expanded coverage.

**Objective:**

To determine whether receipt of first prenatal care services and subsequently receipt of postpartum care through extensions of Emergency Medicaid coverage were associated with increases in diagnosis and treatment of perinatal mental health conditions.

**Design, Setting, and Participants:**

This cohort study used linked Medicaid claims and birth certificate data from 2010 to 2020 with a difference-in-difference design to compare the rollout of first prenatal care coverage in 2013 and then postpartum services in Oregon in 2018 with a comparison state, South Carolina, which did not cover prenatal or postpartum care as part of Emergency Medicaid and only covered emergent conditions and obstetric hospital admissions. Medicaid claims and birth certificate data were linked by Medicaid identification number prior to receipt by the study team. Participants included recipients of Emergency Medicaid who gave birth in Oregon or South Carolina. Data were analyzed from April 1 to October 15, 2023.

**Exposure:**

Medicaid coverage of prenatal care and Medicaid coverage of postpartum care.

**Main Outcomes and Measures:**

The main outcome was the diagnosis of a perinatal mental health condition within 60 days postpartum. Secondary outcomes included treatment of a mood disorder with medication or talk therapy.

**Results:**

The study sample included 43 889 births to Emergency Medicaid recipients who were mainly aged 20 to 34 years (32 895 individuals [75.0%]), multiparous (33 887 individuals [77.2%]), and living in metropolitan areas (32 464 individuals [74.0%]). Following Oregon’s policy change to offer prenatal coverage to pregnant individuals through Emergency Medicaid, there was a significant increase in diagnosis frequency (4.1 [95% CI, 1.7-6.5] percentage points) and a significant difference between states in treatment for perinatal mental health conditions (27.3 [95% CI, 13.2-41.4] percentage points). Postpartum Medicaid coverage (in addition to prenatal Medicaid coverage) was associated with an increase of 2.6 (95% CI, 0.6-4.6) percentage points in any mental health condition being diagnosed, but there was no statistically significant difference in receipt of mental health treatment.

**Conclusions and Relevance:**

These findings suggest that changing Emergency Medicaid policy to include coverage for prenatal and 60 days of postpartum care for immigrants is foundational to improving maternal mental health. Expanded postpartum coverage length, or culturally competent interventions, may be needed to optimize receipt of postpartum treatment.

## Key Points

**Question:**

Is expansion of Medicaid services for prenatal and postnatal care associated with increases in identification and treatment of perinatal mental health conditions for pregnant recent immigrants?

**Findings:**

This cohort study including data from 43 889 births to Emergency Medicaid recipients found that expanding Emergency Medicaid coverage to include prenatal care was associated with significantly increased detection and treatment of perinatal mental health conditions. A similar but smaller association was found with expansion of postpartum coverage.

**Meaning:**

These findings suggest that emergency Medicaid policy changes to include prenatal and postpartum care for immigrants is an important first step to improving maternal mental health among a high-risk population.

## Teaser

This cohort study assesses whether the receipt of first prenatal care services and subsequently postpartum care through extensions of Emergency Medicaid coverage were associated with increases in diagnosis and treatment of perinatal mental health conditions.

## Introduction

Perinatal mental health conditions are pervasive in the US, contributing to a multigenerational cycle of poor health and inequity.^1^ Perinatal mental health conditions, defined as occurring from onset of pregnancy to up to 1 year postpartum, are associated with short-term and long-term health risks for birthing parents and their infants.^1,2^ There are substantial differences in mental health during pregnancy: a large body of observational evidence suggests that people of color (eg, Asian, American Indian or Alaska Native, Black, Hawaiian or Other Pacific Islander, Latino, and multiracial individuals), low-income individuals, adolescents, and individuals with less than a high school education are disproportionately affected by perinatal mental health conditions.^3,4,5,6,7^ Undiagnosed and inadequately treated perinatal mental health conditions are a significant contributor to the US maternal mortality crisis, with suicide being a leading cause of maternal death in the US.^1^

Medicaid policy is a critical lever for maternal mental health care. Medicaid is the largest payer for obstetric care in the US, and thus Medicaid policy and program interventions have a marked effect on maternal health. However, Medicaid policy currently restricts access to care for a population at increased risk for perinatal mental health conditions: low-income, immigrant individuals.^3,4,5,6,7^ The prevalence of postpartum depression alone among immigrant individuals has been reported as ranging from 43% to 60%.^8,9,10^ Immigrant individuals have multiple risk factors for perinatal mental health conditions, including unique barriers to accessing care.^11^

Federal law requires that Medicaid recipients be citizens or permanent residents with greater than 5 years of residence. Full Medicaid benefits are not extended to people who do not meet the citizenship requirements, even if they would qualify on the basis of income.^12^ Instead, these individuals may only receive obstetric delivery coverage through Emergency Medicaid, a federal safety net program covering acute, emergent events and obstetric hospital admissions. No prenatal or postpartum care is covered. This contrasts with traditional Medicaid, which provides full benefits from the diagnosis of pregnancy through a minimum of 60 days postpartum.

While federal Emergency Medicaid does not cover prenatal care, states can extend coverage using state funds or the Children’s Health Insurance Program’s Unborn Child clause. Currently, 18 states, including Oregon, cover prenatal care for immigrants who do not qualify for full Medicaid benefits, while 32 states, including South Carolina, do not.^13^ In 2018, Oregon became one of a handful of states that expanded postpartum care for 60 days for Emergency Medicaid recipients.^14^ South Carolina has not expanded postpartum coverage for the immigrant population.

The objective of this study was to determine the association of 2 policy changes in Oregon that expanded Emergency Medicaid coverage with prenatal and postpartum care outcomes. The first policy change included prenatal care (2010 to 2013) for individuals covered by Emergency Medicaid. The second policy, implemented in 2018, provided postpartum coverage for 60 days for the Emergency Medicaid population in Oregon. We leveraged 9 years of linked birth certificate and Medicaid claims data from Oregon and South Carolina to evaluate the association of prenatal and postpartum care coverage with the diagnosis and treatment of perinatal mental health conditions among the Emergency Medicaid population. We included Oregon and South Carolina because both states have experienced similar growth in their immigrant populations and have comparable immigrant populations in terms of size and country of origin residing in each state. We hypothesized that expanded prenatal and postpartum care would be associated with increased rates of detection of perinatal mental health conditions and treatment.

## Methods

This cohort study was approved by the Oregon Health & Science University institutional review board with a waiver of informed consent because data were deidentified prior to receipt by the study team. We followed the Strengthening the Reporting of Observational Studies in Epidemiology (STROBE) reporting guideline and Reporting of Studies Conducted Using Observational Routinely-Collected Data (RECORD) extension reporting guidelines.

We conducted a retrospective cohort study using linked birth certificate data and Medicaid claims from Oregon and South Carolina. Birth certificate data, which contain a variety of self-reported variables pertaining to pregnancy care and maternal and infant health outcomes, and claims data were linked by Medicaid identification number by each state prior to data transfer to the research team. Our study period was from 2010 to 2020, which allowed for 23 months of births before the policy change expanding prenatal care and 26 months after (October 2010 to December 2015). Our data set also included 35 months of births before the policy change covering postpartum care and 30 months after (January 2016 to October 2020).

### Study Population

Our study population included Emergency Medicaid enrollees ages 12 to 44 years with a live birth between 23 and 44 weeks’ gestation (eFigure in Supplement 1). We used Emergency Medicaid as a measure of immigration status; by federal law, the program’s enrollment is restricted to non–US citizens who meet the financial eligibility requirements for full Medicaid benefits. Consistent with previous studies, women with Emergency Medicaid were identified using program eligibility codes.^15,16^ Given that Emergency Medicaid covers only specific events, no additional enrollment restrictions in the program were applied.

### Outcome Variables

Our primary outcome was the diagnosis of perinatal mental health conditions. Our secondary outcome was the rates of treatment (medication or therapy) received among people diagnosed with a perinatal mental health condition.

We used *International Classification of Diseases, Ninth Revision* (*ICD-9*) and *International Statistical Classification of Diseases and Related Health Problems, Tenth Revision* (*ICD-10*) diagnosis and procedure codes, as well as pharmacy claims to identify births and our outcomes of interest (eTables 1-3 in Supplement 1).^17^ We identified perinatal mental health conditions through the Clinical Classifications Software Refined, which aggregates *ICD-9* and *ICD-10* codes into clinically meaningful categories (eTable 4 in Supplement 1). Treatment of perinatal disorders was defined as receipt of at least 1 prescription for antidepressants, anxiolytics, and other pharmacotherapy indicated for bipolar disorder, or at least 1 encounter for therapy among people with a diagnosis of a perinatal mental health condition.^17,18^ Prenatal care expansion provided health coverage from pregnancy diagnosis through childbirth. We measured perinatal mental health conditions diagnosis and treatment from the start of pregnancy through 60 days postpartum for this policy change. The policy change covering postpartum care ensured additional coverage from birth to 60 days; these individuals also had insurance prenatally. We captured diagnosis and treatment of perinatal mental health conditions in the same way (from pregnancy diagnosis until 60 days following birth) for postpartum care expansion.^19^ We abstracted demographic and clinical information from the birth certificate files and claims data. We included the demographic variables of maternal age, multiparity, race and ethnicity, county of residence (metropolitan, nonmetropolitan, missing), state, maternal tobacco use, and prepregnancy body mass index from birth certificate data. Race and ethnicity were categorized as Asian, Black, Hawaiian Native or Pacific Islander, Latina, White, other (includes the subgroup variable reported as other on the birth certificate), and unknown. We included self-reported information on race and ethnicity from the birth certificate files due to reported racial disparities in perinatal mental health conditions.^20^ We incorporated clinical data relevant to perinatal mental health conditions, including maternal age and rurality, history of mental health disorders, substance abuse, pregnancy complications, preterm birth, and neonatal intensive care unit admission.^21^ Rurality was defined by zip code classification using Rural-Urban Commuting Area Codes.^22^

### Statistical Analysis

To estimate the association of Oregon’s expansion of first prenatal care and then postpartum care with maternal mental health, we used multivariable linear models in a standard difference-in-difference design. We excluded 15 of Oregon’s 36 counties that implemented prenatal care prior to 2013. We include data in our prepolicy period from October 2010 to October 2013 for 21 Oregon counties that expanded prenatal coverage in October 2013. October 2013 to December 2015 served as our postpolicy period for prenatal care expansion.

This design allowed us to compare changes in rates of our outcomes of interest from the prepolicy to postpolicy periods in Oregon and South Carolina populations. We ran separate models for each policy change. The following formula illustrates our model:

Outcome_it_ = β_0_ + β_1_Treatment + β_2_Post + β_3_Treatment × Post + β_4_Age_it_ + β_5_Rural_it_ + e_it_

Where *Outcome_it_* is the outcome of interest for individual *i* at time *t*. *β_1_Treatment* is the treatment group (state) indicator (with Oregon equaling 1 and South Carolina, 0); *β_2_Post*, time relative to intervention (with preintervention equaling 0 and postintervention, 1); and *β_3_Treatment × Post*, the interaction between treatment and time. *β_4_Age_it_* is the covariate maternal age for individual *i* at time *t*; *β_5_Rural_it_*, the indicator for maternal rural residence (with urban equaling 0) for individual *i* at time *t*; and *e_it_*, the error term.

We also used a linear regression model outside the difference in differences design to estimate the association of the treatment group (state) with treatment of perinatal mood disorders during the postpolicy period, since the prepolicy period saw no observations of treatment, with the following formula^23,24^:

Outcome = β_0_ + β_1_Treatment + β2Age + β3Rural + e

Because there might be concern over the use of linear regression for a binary outcome variable, we confirmed our results using logistic models. These models converged and confirmed the direction of the associations (eTable 5 in Supplement 1).

For our model focusing on postpartum coverage, Oregon’s expansion of postpartum care for Emergency Medicaid was implemented statewide on April 1, 2018. We used January 2016 to March 2018 as our prepolicy period, and April 2018 to October 2020 as our postpolicy period for postpartum coverage. We allowed a minimum of 60 days postpartum follow-up in our data set after all births, to correspond with the length of coverage offered under the policy change. We selected covariates for models based on clinical or reported associations with our outcomes. In both models, we adjusted for maternal age and rurality.

We conducted 2-sided tests with an α = .05 and, for secondary outcomes, used a modified Bonferroni correction to account for multiple comparisons.^25^ This approach relies on the assumption that the time trends in outcomes were the same in the 2 states in the prepolicy period (ie, parallel prepolicy trends). We tested the assumption of parallel prepolicy trends for each outcome using linear regression models with bootstrap covariance estimation clustered on the county level.

Standard errors were clustered at the county level. For all of our difference-in-difference models, we conducted 2-sided tests with an α = .05. We use R software version 4.0.3 to conduct our analyses. Data were analyzed from April 1 to October 15, 2023.

## Results

Our final analytic sample included 43 889 births to Emergency Medicaid recipients who were mainly aged 20 to 34 years (32 895 individuals [75.0%]), multiparous (33 887 individuals [77.2%]), and living in metropolitan areas (32 464 individuals [74.0%]), with 22 039 births in the prenatal expansion cohort and 21 850 births in the postpartum expansion cohort (eFigure in Supplement 1). Recipients of Emergency Medicaid expansion differed from individuals who gave birth prior to Emergency Medicaid expansion on a number of clinical and demographic characteristics (Table 1). Within the prenatal expansion cohort, postexpansion recipients were more likely to be older than age 35 years (1742 individuals [20.0%] vs 2049 individuals [15.4%]), less likely to identify as Latina (6786 individuals [77.7%] vs 11 500 individuals [86.4%]), and more likely to be missing rural vs metropolitan residential classification (2415 individuals [27.7%] vs 867 individuals [6.5%]). In the postpartum expansion cohort, postexpansion recipients were also more likely to be older than age 35 years (2623 individuals [23.6%] vs 2356 individuals [21.9%]), less likely to identify as Latina (6310 individuals [56.9%] vs 7196 individuals [66.9%]), and more likely to have an metropolitan residence (8977 individuals [80.9%] vs 8628 individuals [80.2%]). Following postpartum coverage expansion, attendance at a postpartum visit increased from 11.9% to 38.7%. Across all groups, documentation of a history of mental health disorders, an important risk factor for current mental health diagnosis, was rare: in the prenatal expansion cohort, 4 individuals in the pre-expansion group (<0.1%) and 18 individuals in the postexpansion group (0.2%) had a history of mental health disorders, and for the postpartum expansion cohort, 27 individuals in the pre-expansion group (0.3%) and 37 individuals in the postexansion group (0.3%) had a history of mental health disorders. Mood disorder diagnoses were more common in postexpansion groups than the pre-expansion groups in both cohorts (prenatal expansion cohort: 376 individuals [4.3%] vs 260 individuals [2.0%]; postpartum expansion cohort: 737 individuals [6.6%] vs 515 individuals [4.8%]). Our descriptive analyses of types of mental health conditions demonstrated that few recipients had an anxiety diagnosis, with just 0.3% of individuals in the pre-expansion group and 1% in the postexpansion group in the prenatal coverage expansion cohort, and 2% in the both groups in the postpartum coverage expansion cohort (eTable 6 in Supplement 1).

**Table 1.** Demographic and Clinical Characteristics of Emergency Medicaid Births Before and After Policy Expansions, Oregon and South Carolina, 2010 to 2020

Characteristic	Prenatal care expansion			Postpartum care expansion			Total
	Births, No. (%)^a^		*P* value	Births, No. (%)^a^		*P* value	
	Before (n = 13 311)	After (n = 8728)		Before (n = 10 752)	After (n = 11 098)		
Maternal age at birth, y							
<20	896 (6.7)	425 (4.9)	<.001	462 (4.3)	441 (4.0)	<.001	2224 (5.1)
20-34	10366 (77.9)	6561 (75.2)		7934 (73.8)	8034 (72.4)		32 895 (75.0)
≥35	2049 (15.4)	1742 (20.0)		2356 (21.9)	2623 (23.6)		8770 (19.9)
Multiparous	10355 (77.8)	6777 (77.6)	.06	8336 (77.5)	8419 (75.9)	<.001	33 887 (77.2)
Race and ethnicity							
American Indian or Alaska Native	34 (0.3)	34 (0.4)	<.001	42 (0.4)	25 (0.2)	<.001	135 (0.3)
Asian	462 (3.5)	363 (4.2)		632 (5.9)	535 (4.8)		1992 (4.5)
Black	185 (1.4)	138 (1.6)		257 (2.4)	295 (2.7)		875 (1.9)
Hawaiian Native or Pacific Islander	151 (1.1)	135 (1.5)		211 (2.0)	331 (3.0)		828 (1.9)
Latina	11500 (86.4)	6786 (77.7)		7196 (66.9)	6310 (56.9)		31 792 (72.4)
White	347 (2.6)	320 (3.7)		475 (4.4)	470 (4.2)		1639 (3.7)
Other^b^	616 (4.6)	935 (10.7)		1902 (17.7)	2790 (25.1)		6243 (14.2)
Unknown	16 (0.1)	17 (0.2)		37 (0.3)	342 (3.1)		412 (0.9)
County of residence							
Metropolitan	9753 (73.3)	5106 (58.5)	<.001	8628 (80.2)	8977 (80.9)	<.010	32 464 (73.9)
Nonmetropolitan	2691 (20.2)	1207 (13.8)		1974 (18.4)	1941 (17.5)		8083 (18.4)
Missing	867 (6.5)	2415 (27.7)		150 (1.4)	180 (1.6)		3612 (8.2)
Adequate prenatal care (>7 prenatal visits)	11078 (83.2)	7155 (82.0)	.18	8977 (83.5)	9159 (82.5)	.28	36 369 (82.9)
Mood disorder diagnosis	260 (2.0)	376 (4.3)	<.001	515 (4.8)	737 (6.6)	<.001	1888 (4.3)
History of mental health disorder	4 (0.0)	18 (0.2)	<.001	27 (0.3)	37 (0.3)	<.001	86 (0.2)
Prenatal substance use disorder diagnosis	75 (0.6)	99 (1.1)	<.001	278 (2.6)	307 (2.8)	<.001	759 (1.7)
Pregnancy complications^c^	1887 (14.2)	1510 (17.3)	<.001	2016 (18.8)	2288 (20.6)	<.001	7701 (17.5)
Preterm birth (<37 wk gestation)	1011 (7.6)	720 (8.2)	<.001	923 (8.6)	968 (8.7)	.04	3622 (8.2)
Cesarean delivery	3702 (27.8)	2411 (27.6)	.12	3070 (28.6)	3251 (29.3)	.01	12 434 (28.3)
Fetal anomaly	42 (0.3)	73 (0.8)	<.001	142 (1.3)	176 (1.6)	<.001	433 (0.9)

^a^ Individual variable denominators differ depending on missingness.

^b^ Includes the subgroup variable reported as other on the birth certificate.

^c^ Pregnancy complications include gestational diabetes, prepregnancy diabetes, gestational hypertension, and prepregnancy hypertension.

Our difference-in-difference models rely on the assumption of parallel trends in outcomes prior to the policy change. We qualitatively examined raw trends over time for both models, and quantitatively assessed parallel pre-expansion trends (Figure and Tables 2 and Table 3). We did not find any significant difference in our outcomes prior to the policy changes for all outcomes.

**Figure.**
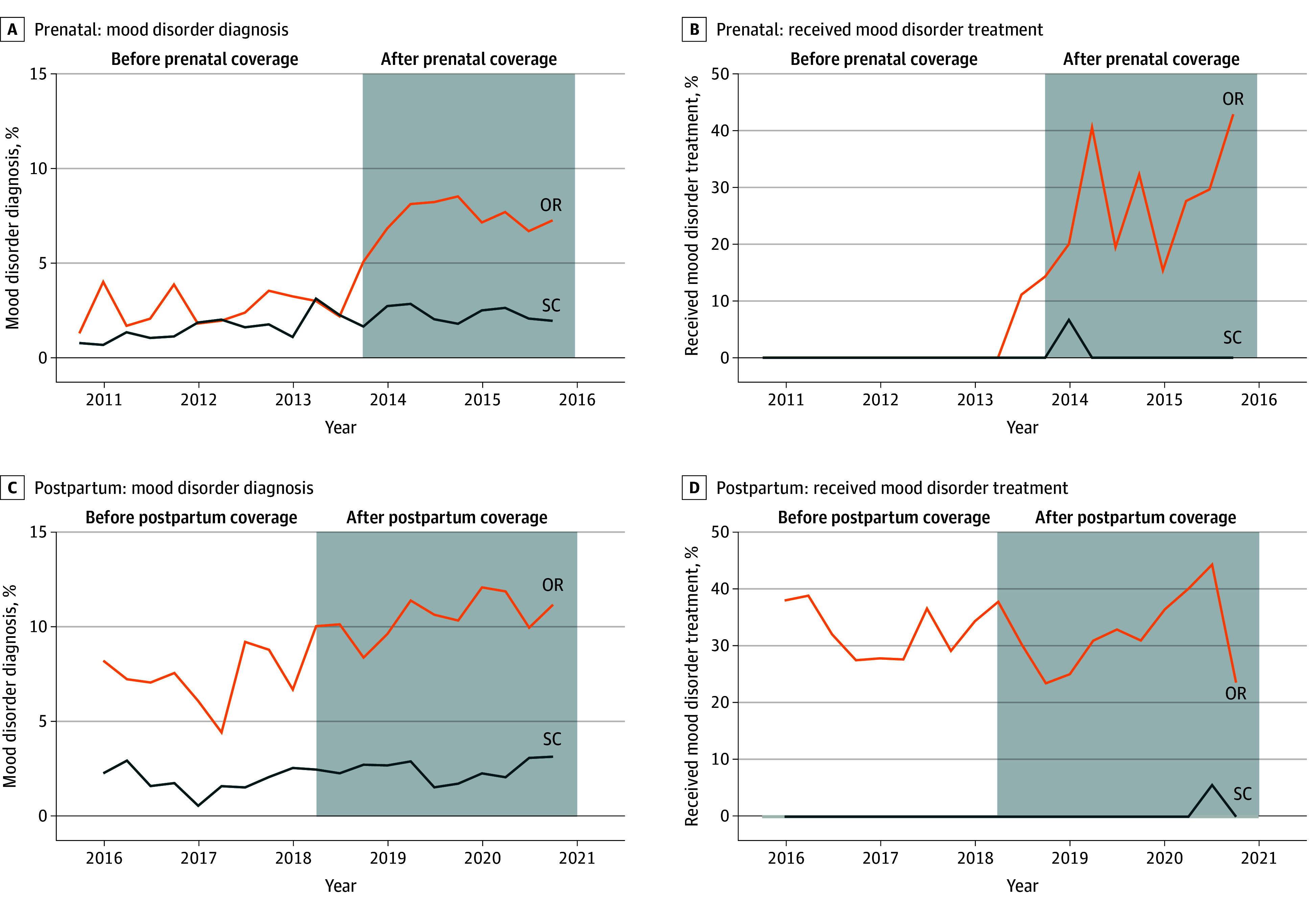
Trend Estimates of Mental Health Condition Diagnosis and Treatment During the Prenatal Period and Within 60 Days Postpartum Among Emergency Medicaid Recipients Before and After Prenatal Coverage Expansion OR indicates Oregon; SC, South Carolina.

**Table 2.** Changes in Mental Health Diagnosis and Treatment Among the Emergency Medicaid Population Following Expansion of Prenatal Care, 2010 to 2015

Outcome	Treatment group (Oregon)			Comparison group (South Carolina)			DID estimate, percentage points (95% CI)^a^
	Women, No. (%)		Difference, percentage points	Women, No. (%)		Difference, percentage points	
	Pre-expansion (n = 5538)	Postexpansion (n = 3569)		2011-2013 (n = 7773)	2014-2015 (n = 5159)		
Mental health diagnosis^b^	142 (2.6)	260 (7.3)	4.7	118 (1.5)	116 (2.2)	0.7	4.1 (1.7-6.5)
Mental health treatment^c^	1 (0.7)	72 (27.0)	26.3	0	1 (0.9)	0.9	26.5 (15.5-37.5)

Abbreviation: DID, difference-in-differences.

^a^ Difference-in-difference estimates adjust for maternal age.

^b^ Pre-expansion test of parallel trends: *P* = .28.

^c^ Includes talk therapy or medications. Pre-expansion test of parallel trends: *P* = .16.

**Table 3.** Changes in Mental Health Diagnosis and Treatment Among the Emergency Medicaid Population Following Expansion of Postpartum Care, 2016-2020

Outcome	Treatment (Oregon)			Comparison (South Carolina)			DID estimate, percentage points (95% CI)^a^
	Women, No. (%)		Difference, percentage points	Women, No. (%)		Difference, percentage points	
	Pre-expansion (n = 5880)	Postexpansion (n = 5890)		2016-2017 (n = 4872)	2018-2020 (n = 5208)		
Mental health diagnosis^b^	426 (7.2)	612 (10.4)	3.2	89 (1.8)	125 (2.4)	0.6	2.6 (0.6 to 4.6)
Mental health treatment^c^	140 (32.9)	202 (33.0)	0.1	0	1 (0.8)	0.8	0.1 (−7.5 to 7.7)

Abbreviation: DID, difference-in-differences.

^a^ Difference-in-difference estimates adjust for maternal age.

^b^ Pre-expansion test of parallel trends: *P* = .89.

^c^ Includes talk therapy or medications.

The expansion of prenatal Medicaid coverage for Emergency Medicaid recipients was associated with an increase of 4.1 (95% CI, 1.7 to 6.5) percentage points in any mental health condition being diagnosed prenatally through 60 days postpartum (Table 2 and Figure, A). Among individuals with a mental health diagnosis during the postexpansion period for the prenatal cohort, state was associated with an increase of 27.3 (95% CI, 13.2 to 41.4) percentage points in receipt of any mental health treatment (medication or therapy). We observed more modest gains with postpartum expansion of Emergency Medicaid. Postpartum Medicaid coverage (in addition to prenatal Medicaid coverage) was associated with an increase of 2.6 (95% CI, 0.6 to 4.6) percentage points in any mental health condition being diagnosed (Table 2 and Figure, C). No statistically significant increase in receipt of mental health treatment was observed (Figure, D).

Sensitivity analyses that restricted the sample to Latina individuals with Emergency Medicaid found similar increases in mood disorder diagnosis (4.2 [95% CI, 1.8 to 6.6] percentage points) (eTable 7 in Supplement 1) and mood disorder treatment (27.4 [95% CI, 17.0 to 37.8] percentage points) associated with the expansion for prenatal care. However, among Latina individuals only, we found that neither mood disorder diagnosis (2.4 [95% CI, −0.1 to 4.9] percentage points) or mood disorder treatment (0.0 [95% CI, 0.0 to 0.0] percentage points) had a statistically significantly change associated with the expansion of postpartum Medicaid coverage (eTable 8 in Supplement 1).

## Discussion

Our cohort study found mental health benefits associated with expanding Medicaid coverage during pregnancy among low-income migrants. Expansion of prenatal care coverage was associated with a 4.1–percentage point increase in the identification of perinatal mental health conditions and a 26.5–percentage point increase in people receiving treatment. We identified a more modest, but still significant mental health benefit associated with expansion of postpartum care (2.6 percentage point increase in diagnosis; no significant increase in treatment).

The US has one of the highest rates of maternal mortality and morbidity among developed nations; people with low income and communities of color are disproportionately affected. In the US, 20% of all maternal deaths are due to suicide, and individuals with perinatal mental health conditions are 50% more likely to experience severe maternal morbidity than those without a mental health condition.^26^

We found that prenatal care expansion was associated with a greater increase in diagnosis and treatment of mental health condition than postpartum care expansion. This finding may reflect that 60 days postpartum is an inadequate amount of time to capture new diagnoses of perinatal mental health conditions. While symptoms of mental health conditions usually begin within a few weeks of giving birth, postpartum mental health conditions can occur any time in the first year postpartum. This finding may also be due to low postpartum visit attendance by Emergency Medicaid recipients despite the policy change. Following postpartum care expansion, visit attendance increased from 11.9% to 38.7%, but still lagged markedly behind the 58.8% of traditional Medicaid recipients receiving care.^27^ Efforts are needed to identify and remove barriers to postpartum visit attendance among Emergency Medicaid recipients. We did not identify a significant increase in treatment for perinatal mental health conditions following expansion of postpartum care. This finding may be due to small sample size, but also it may reflect that Emergency Medicaid recipients were only followed-up for 60 days postpartum, to align with the policy change.

It is important to note that expanded coverage alone was insufficient to address the stark differences in mental health diagnoses observed between traditional and Emergency Medicaid recipients. We examined the incidence of perinatal mental health conditions at 4 time points: before and after prenatal care expansion and before and after postpartum care expansion. At each of these time points, meaningful differences in both diagnosis and treatment by type of Medicaid were observed. Previous research has estimated a 20% occurrence of postpartum depression alone among immigrant women.^11^ While this may reflect baseline differences in our populations, it is probable that differences in screening also drive our finding.

The most widely used screening tool in the US is the Edinburgh Postnatal Depression Scale, which is a self-reported, 10-item Likert scale.^11^ The validity of the Edinburgh Postnatal Depression Scale in identifying depression among immigrant women, particularly when an interpreter is used, has been questioned.^28^ Previous studies have demonstrated multiple barriers associated with screening for mental health conditions among the immigrant population in the US, including poor translations of screening tools, difficulty with interpretative services, lower health literacy, and the use of tools developed in Western societies and applied to culturally diverse immigrant populations.^29,30^

Poor maternal mental health has multigenerational implications. Parental depression in the postpartum period is associated with less healthy infant sleep and infant feeding practices and decreased positive enrichment activities with the child (eg, reading, singing, playing).^31^ Maternal postpartum depression has been negatively associated with language development markers (eg, vocal productivity scores and conversational turns).^31^

### Limitations

Our study has some limitations. Use of administration data means our data are subject to errors in coding. However, our study is strengthened by the use of 2 distinct data sources, Medicaid claims and birth certificate data, which allowed us to corroborate health outcomes and improve the demographic information available. We used data from 2 states, Oregon and South Carolina, which may limit our generalizability to other areas. We do not know whether people giving birth were screened for mental health conditions in their native language; failure to do so would decrease the sensitivity of detection and bias our results toward the null.

## Conclusions

Our cohort study found a substantial disparity in diagnosis of mental health disorders during pregnancy among immigrant individuals. This inequity has multigenerational consequences. Poor perinatal mental health is a meaningful contributor to the US’s maternal health crisis. Further research should focus on validation of culturally appropriate tools for screening for mental health conditions among immigrant populations and identifying interventions to best support immigrant individuals needing care.

## Notes

Supplement 1. **eFigure.** Study Recruitment Flowchart **eTable 1.** Codes for Identifying Hospital Births in Medicaid Claims **eTable 2.** Procedure Codes for Therapy Encounters **eTable 3.** National Drug Codes for Pharmaceutical Claims **eTable 4.** *ICD-9* and *ICD-10* Codes for Mood Disorder Categories **eTable 5.** Sensitivity Analysis of Logistic Regression Model Estimates Compared With Linear Regression **eTable 6.** Perinatal Mental Health Diagnoses Among the Emergency Medicaid Population in Oregon and South Carolina Following Expansion of Prenatal Coverage and Postpartum Coverage **eTable 7.** Changes in Mood Disorder Diagnosis and Treatment Among the Emergency Medicaid Population Following Expansion of Postpartum Care Among Latina Individuals Only, 2016-2020 **eTable 8.** Changes in Mood Disorder Diagnosis and Treatment Among the Emergency Medicaid Population Following Expansion of Postpartum Care Among Latina Individuals Only, 2016-2020

Supplement 2.
Data Sharing Statement
