# Supplementary material for: Diagnosis of Perinatal Mental Health Conditions Following Medicaid Expansion to Include Low-Income Immigrants
Source: JAMA Netw Open. 2024 Feb 20;7(2):e240062. doi: 10.1001/jamanetworkopen.2024.0062 (PMC10879944; doi:10.1001/jamanetworkopen.2024.0062)
Supplement: Supplement 1. — eFigure. Study Recruitment Flowchart eTable 1. Codes for Identifying Hospital Births in Medicaid Claims eTable 2. Procedure Codes for Therapy Encounters eTable 3. National Drug Codes for Pharmaceutical Claims eTable 4. ICD-9 and ICD-10 Codes for Mood Disorder Categories eTable 5. Sensitivity Analysis of Logistic Regression Model Estimates Compared With Linear Regression eTable 6. Perinatal Mental Health Diagnoses Among the Emergency Medicaid Population in Oregon and South Carolina Following Expansion of Prenatal Coverage and Postpartum Coverage eTable 7. Changes in Mood Disorder Diagnosis and Treatment Among the Emergency Medicaid Population Following Expansion of Postpartum Care Among Latina Individuals Only, 2016-2020 eTable 8. Changes in Mood Disorder Diagnosis and Treatment Among the Emergency Medicaid Population Following Expansion of Postpartum Care Among Latina Individuals Only, 2016-2020 [file jamanetwopen-e240062-s001.pdf]

Supplemental Online Content

Rodriguez MI, Martinez-Acevedo A, Kaufman M, Nacev EC, Mackiewicz-Seghete K, McConnell KJ. Diagnosis of perinatal mental health conditions following Medicaid expansion to include low-income immigrants. *JAMA Netw Open*. 2024;7(2):e240062.
doi:10.1001/jamanetworkopen.2024.0062

eFigure. Study Recruitment Flowchart

eTable 1. Codes for Identifying Hospital Births in Medicaid Claims

eTable 2. Procedure Codes for Therapy Encounters

eTable 3. National Drug Codes for Pharmaceutical Claims

eTable 4. *ICD-9* and *ICD-10* Codes for Mood Disorder Categories

eTable 5. Sensitivity Analysis of Logistic Regression Model Estimates Compared With Linear Regression

eTable 6. Perinatal Mental Health Diagnoses Among the Emergency Medicaid Population in Oregon and South Carolina Following Expansion of Prenatal Coverage and Postpartum Coverage

eTable 7. Changes in Mood Disorder Diagnosis and Treatment Among the Emergency Medicaid Population Following Expansion of Postpartum Care Among Latina Individuals Only, 2016-2020

eTable 8. Changes in Mood Disorder Diagnosis and Treatment Among the Emergency Medicaid Population Following Expansion of Postpartum Care Among Latina Individuals Only, 2016-2020

This supplemental material has been provided by the authors to give readers additional information about their work.

© 2024 Rodriguez MI et al. *JAMA Network Open.*

eFigure. Study Recruitment Flowchart

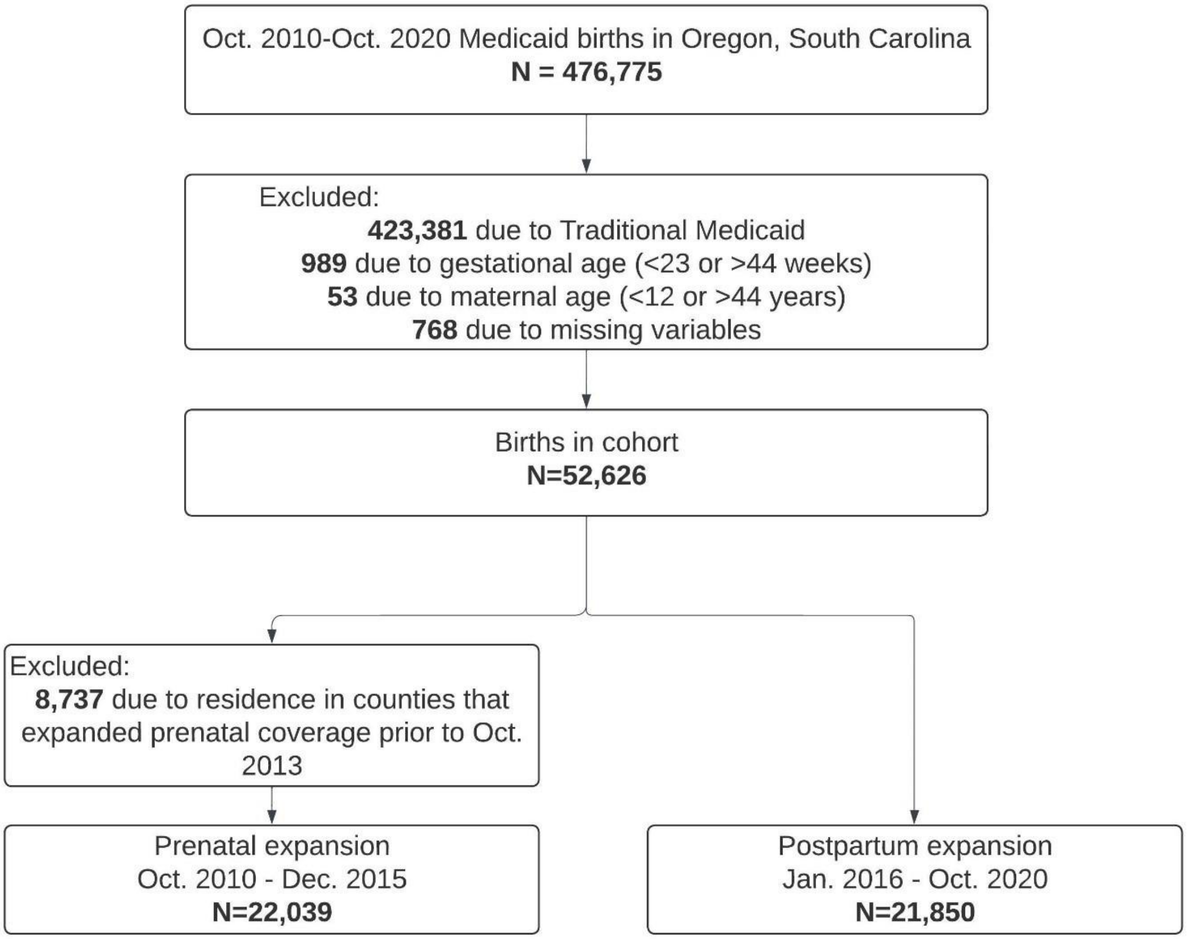

eTable 1. Codes for Identifying Hospital Births in Medicaid Claims

Category	Code Type	Codes
Hospital Birth Event	Diagnosis-related Group (DRG)	765, 766, 767, 768, 774, 775, 783, 784, 785, 786, 787, 788, 796, 797, 798,

© 2024 Rodriguez MI et al. *JAMA Network Open.*

eTable 2. Procedure Codes for Therapy Encounters

**Category**	**Code Type**	**Codes**
Therapy	Common Procedure Terminology (CPT)	90832, 90833, 90834, 90836, 90837, 90838, 90839, 90840, 90846, 90847, 90849, 90853, 90863, G0176, H2032

eTable 3. National Drug Codes for Pharmaceutical Claims

Pharmaceutical	National Drug Code (NDC)	93029001, 93029005, 93029019, 93029093, 173017855, 247196100, 247196114, 247196130, 247196160, 378043501, 378043505, 781106401, 781106410, 904591361, 904612961, 21695001830, 21695001860, 21695001890, 23490517203, 23490517206, 49999034900, 49999034930, 49999034990, 51079094401, 51079094420, 52959065500, 52959065502, 52959065530, 52959065540, 52959065560, 52959065590, 54569539100, 54569539102, 54569539103, 54868455000, 54868455001, 54868455002, 55045319506, 55045319508, 55289073330, 55289073360, 55887015101, 55887015120, 55887015130, 55887015160, 55887015182, 55887015190, 58016058400, 58016058402, 58016058403, 58016058410, 58016058412, 58016058415, 58016058420, 58016058425, 58016058430, 58016058440, 58016058450, 58016058460, 58016058470, 58016058473, 58016058480, 58016058489, 58016058490, 58864080890, 60429094701, 60429094705, 60505015701, 60505015705, 60505015709, 63629264201, 63874061601, 63874061604, 63874061610, 63874061612, 63874061615, 63874061620, 63874061625, 63874061628, 63874061630, 63874061640, 63874061650, 63874061660, 63874061670, 63874061672, 63874061674, 63874061677, 63874061680, 63874061690, 68001019800, 68115041200, 68387035360, 93550101, 173094755, 185041001, 185041005, 185041060, 378341101, 378341105, 591085860, 591354005, 591354060, 10370015906, 13668043060, 16590003630, 16590003660, 16714033401, 16714033402, 16714033403, 21695029560, 23490923103, 33358005360, 47335073613, 47335073686, 47335073688, 49999034960, 51079039101, 51079039120, 52959080560, 54569556800, 58016024000, 58016024002, 58016024003, 58016024030, 58016024060, 58016024090, 58864071530, 58864084060, 59746031501, 63874092601, 63874092604, 63874092610, 63874092628, 63874092630, 63874092660, 63874092690,

© 2024 Rodriguez MI et al. *JAMA Network Open.*

		64679010102, 64679010103, 66105048001, 66105048002, 66105048003, 66105048006, 66105048015, 67544046482, 67544049982, 67767017160, 68084047001, 68084047011, 68084069701, 68084069711, 93550201, 93570301, 173013555, 173055601, 173055602, 185041501, 185041505, 185041552, 185041560, 247167500, 247167511, 247167514, 247167560, 247167710, 247167760, 247207730, 247207760, 378341201, 378341205, 378552101, 591083925, 591083960, 591354105, 591354125, 591354160, 591354360, 591354376, 781152960, 781516960, 10370016006, 10370016010, 10370016050, 13668043125, 13668043160, 16590003830, 16590003860, 16714033501, 16714033502, 16714033503, 21695001930, 21695001960, 21695057730, 23490768903, 23490768906, 23490923006, 43063024330, 43063050330, 47335073713, 47335073786, 47335073788, 49999038130, 49999096530, 49999096560, 51079039201, 51079039220, 52959028530, 52959028560, 52959048730, 52959080630, 52959080660, 54569556900, 54569616000, 54868402500, 54868489200, 54868489201, 55045263108, 55045319706, 55289090530, 55887049630, 55887049690, 55887066330, 55887066360, 55887066390, 58016002400, 58016002430, 58016002460, 58016002490, 58016059900, 58016059930, 58016059960, 58016059990, 58016088100, 58016088102, 58016088103, 58016088130, 58016088160, 58016088190, 58864062530, 58864062560, 58864084030, 59746031601, 59746031660, 63874078020, 64679010502, 64679010503, 64679010504, 65084041413, 66105048101, 66105048102, 66105048103, 66105048106, 66105048115, 66336089730, 66336089760, 67544029930, 67544054730, 67767011760, 67767013305, 67767013325, 67767013360, 68084047101, 68084047111, 68084070825, 68084070895, 68115044530, 93535005, 93535056, 115681102, 115681108, 115681110, 173073001, 173073002, 187073030, 187073090, 378200877, 591333105, 591333119, 591333130, 10370010100, 10370010103, 10370010150, 16590052630, 16590052660, 16590052690, 18837017530, 21695013715, 21695013730, 21695013745, 21695027930, 42291018230, 42291018250, 42291018290, 43063038790, 45963014130, 45963014190, 49999077430, 49999077460, 49999077490, 51079004701, 51079004720, 52959082060, 54868592700, 55289092215, 55289092230, 55887028430, 55887028460, 55887028490, 58016003100, 58016003130, 58016003160, 58016003190, 60429035705, 60429035730, 60429035790, 60687014601, 60687014611, 63739044910, 64455073030, 64455073090, 64679010201, 64679010202, 67767014130, 67767014190, 67801043330, 68001026104, 68084025101, 68084025111, 115544513, 173072200, 185111160, 378341301,

© 2024 Rodriguez MI et al. *JAMA Network Open.*

		378341305, 591354260, 10370016106, 13668043260, 16714033601, 21695002060, 43063018360, 47335073886, 64679010702, 64679010703, 67767013560, 93535105, 93535156, 173073101, 187073130, 247235600, 247235630, 378200905, 490016300, 490016330, 490016360, 490016390, 591333205, 591333230, 10370010200, 10370010203, 10370010250, 16590024630, 16590024660, 16590024690, 18837017630, 21695013815, 21695013830, 21695057830, 21695064130, 35356008730, 35356008760, 35356008790, 42291018330, 42291018350, 43063038890, 45963014205, 45963014230, 45963014290, 49999044315, 49999044330, 51079010901, 51079010903, 52959086930, 54569559900, 54569596900, 54868493500, 54868573600, 54868573601, 55289090015, 55289090030, 55887028330, 55887028360, 55887028390, 58016067100, 58016067102, 58016067103, 58016067130, 58016067160, 58016067190, 60429035805, 60429035830, 60429035890, 63739045010, 64455073130, 67767014205, 67767014230, 67767014290, 68001026403, 68001026404, 68001026405, 68084025211, 68084025221, 68382035405, 68382035406, 49909001030, 93028001, 93028005, 93028019, 93028093, 173017755, 378043301, 378043305, 781105301, 781105310, 904609361, 16590003730, 16590003760, 21695001700, 21695001760, 23490517303, 23490923203, 43063028430, 43063028498, 49999091830, 51079094301, 51079094320, 51079094330, 51079094356, 52959089860, 54868413400, 55887065090, 55887065092, 58016072200, 58016072202, 58016072203, 58016072220, 58016072230, 58016072240, 58016072250, 58016072260, 58016072270, 58016072273, 58016072280, 58016072289, 58016072290, 58864079430, 60429094601, 60429094605, 60505015801, 60505015805, 60505015809, 63874056730, 63874056760, 68001019900, 68387035030, 68387035060, 24581030, 187581030, 24581130, 187581130, 24581230, 187581230, 456111030, 456112030, 456114030, 456110031, 456110130, 64764056030, 64764073030, 64764058030, 64764075030, 64764055030, 64764072030, 30698003201, 71035060, 43386036021, 59762011901, 187045302, 39506002260, 49502042060, 51079013701, 51079013720, 51079088701, 51079088703, 60429001960, 60505005501, 60505005502, 67253070006, 378929010, 378929091, 51862014606, 59772090810, 59772090820, 60429017660, 60505343803, 60505343808, 60763010202, 60763010203, 62033010202, 62033010203, 7447120, 591559001, 24987044710, 49884003201, 59212044710, 64980018301, 49502090230, 49502090030, 49502090130, 93102406, 172433249, 591076460, 16590016630, 16590016660, 16590016690, 21695017460, 23490657701, 49884091702, 49884091705,

© 2024 Rodriguez MI et al. *JAMA Network Open.*

		58016010030, 58016010060, 58016010090, 63874054214, 63874054220, 63874054230, 63874054260, 93711306, 172433349, 21695017560, 49884091802, 49884091805, 58016054600, 58016054630, 58016054660, 58016054690, 63874054020, 63874054030, 63874054060, 93102506, 172433449, 21695017630, 21695017660, 23490657901, 23490657902, 23490657903, 49884091902, 49884091905, 58016033600, 58016033630, 58016033660, 58016033690, 93102606, 172433549, 591076760, 21695017730, 21695017760, 42291062760, 49884092002, 49884092005, 55111014260, 93717801, 172434349, 49884091602, 49884091605, 182126000, 182126089, 247181304, 247181308, 247181330, 378347201, 378347210, 440857630, 555049002, 555049004, 591559901, 591559910, 603616102, 603616104, 603616116, 603616120, 603616121, 603616128, 603616132, 904399161, 904655561, 13668033101, 13668033105, 16590023230, 16590023245, 16590023260, 16590023290, 21695013430, 21695013460, 21695013490, 23490640501, 23490640502, 23490640509, 33358034530, 33358034590, 42291083410, 43353043530, 49999015600, 49999015614, 49999015620, 49999015630, 49999015660, 50111043401, 50111043402, 50111043403, 51079042801, 51079042819, 51079042820, 52959014030, 52959014060, 52959014090, 53489051101, 54569199900, 54569199901, 54569199902, 54569199903, 54868122300, 54868122301, 55045172400, 55045172401, 55045172406, 55045172408, 55045172409, 55289022330, 55289022360, 55289022390, 55887025620, 57866468801, 57866468802, 57866468803, 57866468804, 58016086200, 58016086202, 58016086203, 58016086212, 58016086215, 58016086220, 58016086230, 58016086250, 58016086260, 58016086273, 58016086289, 58016086290, 58864078330, 60429018801, 60429018805, 60429018830, 60429018860, 60429018890, 60429098801, 60429098805, 60429098830, 60505265400, 60505265401, 60505265405, 60505265407, 60760043430, 60760043460, 63629321001, 63629321002, 63739024610, 63874053701, 63874053704, 63874053710, 63874053712, 63874053714, 63874053715, 63874053720, 63874053730, 63874053740, 63874053760, 63874053790, 65243032703, 66336001430, 66336001460, 67544016230, 67544033130, 68084012501, 68387016630, 68387016660, 68645045270, 247104103, 247104107, 247104130, 378347301, 378347310, 555073202, 555073204, 13668033201, 21695013560, 23490640601, 23490640602, 23490640606, 23490640609, 33358034630, 33358034660, 33358034690, 42291083550, 49999091330, 50111044101, 50111044102, 52959089430, 52959089460, 53489051701, 54569373201, 54569373202,

© 2024 Rodriguez MI et al. *JAMA Network Open.*

		54569373203, 54868195901, 54868195902, 54868195904, 55045250900, 55045250901, 55045250906, 55045250908, 55045250909, 55289006014, 55289006030, 55864070830, 55887087130, 58016088000, 58016088012, 58016088015, 58016088020, 58016088030, 59772317101, 60429023001, 60429023005, 60429023030, 60429023090, 60429093001, 60429093005, 60429093030, 60429093090, 60505265501, 60505265505, 60505265507, 60505408903, 61392049130, 63629286701, 63629286702, 63629286703, 63874077501, 63874077512, 63874077515, 63874077520, 63874077530, 66336083814, 68084060801, 68084060811, 68387016030, 43595008003, 247104230, 378347401, 555073302, 13668033301, 42291083601, 58016070100, 58016070112, 58016070115, 58016070120, 58016070130, 58016070150, 58016070160, 58016070190, 60505265901, 43595008103, 182125900, 182125989, 247067300, 247067307, 247067310, 247067314, 247067330, 247067360, 247067390, 378347101, 440857520, 555048902, 555048904, 591560001, 591560010, 603614721, 603614732, 603616002, 603616013, 603616016, 603616020, 603616021, 603616028, 603616032, 904399061, 904521960, 904655461, 10544034630, 10544034660, 13668033001, 13668033005, 16590023130, 16590023160, 16590023190, 21695013330, 21695013360, 21695013390, 23490640701, 23490640702, 23490640703, 23490640704, 33358034420, 33358034430, 33358034460, 42291083310, 49999034300, 49999034315, 49999034330, 49999034360, 50111043301, 50111043302, 50111043303, 51079042701, 51079042719, 51079042720, 52959037815, 52959037820, 52959037830, 52959037860, 52959037890, 53489051001, 54569147000, 54569147001, 54569147006, 54569147008, 54569147009, 54868012200, 54868012202, 55045171500, 55045171501, 55045171502, 55045171503, 55045171507, 55045171508, 55045171509, 55045375708, 55289006407, 55289006414, 55289006430, 55289006460, 55289006490, 57866471501, 57866471502, 57866471503, 57866471504, 58016026300, 58016026302, 58016026303, 58016026310, 58016026312, 58016026315, 58016026320, 58016026328, 58016026330, 58016026360, 58016026373, 58016026389, 58016026390, 58864002501, 58864002514, 58864002530, 58864002560, 60429018705, 60429018730, 60429098701, 60429098705, 60429098730, 60429098760, 60429098790, 60505265300, 60505265301, 60505265305, 60505265307, 60760044030, 60760044060, 63629151301, 63629151302, 63629151303, 63739024510, 63874056001, 63874056004, 63874056010, 63874056012, 63874056014, 63874056015, 63874056020, 63874056028, 63874056030, 63874056040, 63874056060, 63874056090,

© 2024 Rodriguez MI et al. *JAMA Network Open.*

		66336062028, 66336062030, 66336062060, 67544031615, 68084012401, 68387016530, 68387016590, 68645045170, 187380510, 378021101, 378021105, 49884096101, 54569017300, 187380610, 378027701, 378027705, 49884096201, 54868042600, 378033001, 378033005, 378004201, 378044201, 378044205, 49999055130, 378057401, 378057405, 378007301, 2323230, 93550656, 781219231, 49884025211, 2323030, 93550356, 781219531, 49884027711, 2323101, 2323130, 2323133, 93550456, 781219131, 49884025011, 2323401, 2323430, 2323433, 93550756, 781219431, 49884025311, 2323301, 2323330, 2323333, 93550556, 781219331, 49884025111, 8122201, 8122214, 8122230, 8122250, 44183089031, 44183089090, 47335080483, 49884037509, 49884037511, 63304019230, 63304019290, 65224089031, 65224089090, 8121030, 8121101, 8121114, 8121130, 8121150, 35356049130, 43063026230, 44183088031, 44183088090, 47335074781, 47335074783, 49884037409, 49884037411, 63304019130, 63304019190, 65224088031, 65224088090, 2323501, 2323533, 2323560, 93754206, 228289006, 904636661, 904645261, 13668010960, 18837020760, 21695065760, 47335038186, 47335038188, 51991074605, 51991074606, 51991074690, 54868521500, 54868521501, 54868521502, 55111060860, 57237001760, 58016073500, 58016073530, 58016073560, 58016073590, 60429016460, 60505299506, 66993007560, 66993066260, 68001025506, 68084067521, 68180029403, 68180029406, 68180029407, 68382038514, 69097029703, 2324001, 2324030, 2324033, 2324090, 93754356, 228289103, 228289150, 904636761, 904645361, 13668011005, 13668011030, 16590006630, 16590006660, 16590048230, 16590048260, 16590048272, 16590048290, 21695014515, 21695014530, 33358009830, 33358009860, 47335038281, 47335038283, 49999061830, 49999061860, 51991074710, 51991074790, 52959017330, 54569594101, 54569870300, 54569870301, 54868531500, 55111060930, 55111060990, 55289003621, 55289003630, 57237001830, 57237001890, 57237001899, 58016073200, 58016073230, 58016073260, 58016073290, 60429016530, 60429016590, 60505299603, 66993007630, 66993066305, 66993066330, 68001025604, 68001025605, 68071044415, 68071044460, 68071131200, 68084068301, 68180029503, 68180029506, 68180029509, 68382038606, 68382038616, 68387055430, 68387055460, 69097029802, 27437029806, 68180029706, 2323701, 2323704, 2323730, 2323733, 2327001, 2327004, 2327030, 2327033, 93754456, 228289203, 228289296, 904636861, 904645461, 12280017160, 13668011105, 13668011130, 16590006730, 16590006760, 16590048330, 16590048360, 16590048372, 16590048390, 18837003590,

© 2024 Rodriguez MI et al. *JAMA Network Open.*

		21695014615, 33358009930, 33358009960, 47335038318, 47335038383, 49999061930, 49999061960, 51991074810, 51991074890, 52959089230, 52959089260, 54569567800, 54569870200, 54868519200, 55045369703, 55111061010, 55111061030, 55289002821, 55289002830, 57237001930, 57237001990, 57237001999, 58016074200, 58016074230, 58016074260, 58016074290, 60429016610, 60429016630, 60505299703, 63629334001, 63874125303, 66993007730, 66993066405, 66993066430, 68001025704, 68071044515, 68071044560, 68071131300, 68084069201, 68180029603, 68180029606, 68180029609, 68382038706, 68387055330, 68387055360, 69097029902, 456221230, 456222030, 456220228, 456224030, 456228030, 93738301, 378488501, 603615121, 603615125, 13811067610, 16714031501, 21695072060, 23155025001, 23155025009, 42291089650, 42291089690, 50268080215, 55111054990, 57237017601, 57237017690, 57664039688, 59762348402, 62332001231, 65162030509, 65862040801, 65862040820, 68001015600, 68084090525, 68084090595, 68382010101, 68382010110, 8083602, 8083603, 8083620, 8083621, 8083622, 93738605, 93738656, 93738698, 378513305, 904624861, 904647061, 13668002001, 13668002005, 13668002030, 13668002074, 13668002090, 16590008760, 18837004890, 21695004715, 21695004745, 33358012330, 33358012360, 33358012390, 49999059915, 49999059930, 49999059960, 51079013601, 51079013604, 52959038830, 52959038860, 54569523100, 54569622700, 54868425200, 54868425201, 54868425202, 54868425203, 54868425204, 55045336808, 55111045505, 55111045530, 55111045590, 55289089730, 55887019390, 58016061600, 58016061630, 58016061660, 58016061690, 58864075630, 59762018201, 59762018202, 59762018203, 59762018204, 60505378003, 60505378009, 63739051210, 63739063310, 64679071801, 64679071804, 64679071805, 65862052930, 65862052990, 65862069701, 65862069705, 65862069730, 65862069790, 67544018930, 67801031203, 68071041215, 68071041230, 68071041260, 68084048601, 68084048611, 68084071301, 68382003606, 68382003610, 68382003616, 68387034915, 68387034930, 131326732, 131326746, 41616075881, 41616075883, 65580030303, 65580030309, 131326832, 131326846, 65580030403, 65580030409, 93019901, 378488101, 603615621, 13811067210, 16714031101, 21695071560, 23155024601, 23155024609, 42291089290, 50268079815, 55111054590, 57237017201, 57237017290, 57664039288, 59762348003, 62332000831, 65162030009, 65862040401, 65862040460, 68001015700, 68084089625, 68084089695, 68382001801, 68382001810, 8083702, 8083703, 8083720, 8083721, 8083722, 93738405,

© 2024 Rodriguez MI et al. *JAMA Network Open.*

		93738456, 93738498, 247185004, 247185008, 247185030, 378513105, 904624661, 904646861, 13411010003, 13411010901, 13411010906, 13411010909, 13411010910, 13668001801, 13668001805, 13668001830, 13668001874, 13668001890, 16590008530, 16590008560, 21695004515, 51079013401, 51079013420, 52959077160, 54569526500, 54868450400, 54868450401, 55111045305, 55111045330, 55111045390, 55289086930, 55887066230, 55887066260, 58016076500, 58016076530, 58016076560, 58016076590, 58864069630, 59762018001, 59762018002, 59762018003, 59762018004, 60505377803, 60505377809, 63739052110, 64679071601, 64679071604, 64679071605, 65862052701, 65862052730, 65862052790, 65862052799, 66105011110, 67801032103, 68071033330, 68084048401, 68084048411, 68084069801, 68382003406, 68382003410, 68382003416, 93738001, 378488201, 603615721, 603615725, 13811067310, 16714031201, 21695071630, 23155024701, 23155024709, 42291089390, 43063029230, 43353037053, 43353044153, 50268079915, 51079048001, 51079048020, 54868405500, 54868405501, 55111054605, 55111054690, 57237017301, 57237017390, 57664039388, 58016000400, 58016000430, 58016000460, 58016000490, 59762348102, 62332000931, 65162030209, 65862040501, 65862040560, 65862040590, 68001015800, 68084033001, 68084033011, 68084084401, 68084084411, 68382001901, 68382001910, 131326532, 131326546, 41616076081, 41616076083, 65580030103, 65580030109, 93738101, 378488301, 603614921, 13811067410, 16714031301, 23155024801, 23155024809, 42291089490, 50268080015, 55111054790, 57237017401, 57237017490, 57664039488, 59762348202, 62332001031, 65162030609, 65862040601, 65862040630, 65862040690, 68001015900, 68084090025, 68084090095, 68382002001, 68382002010, 8083302, 8083303, 8083320, 8083321, 8083322, 93738505, 93738556, 93738598, 247233730, 247233760, 247233790, 378513205, 904624761, 904646961, 13411011001, 13411011003, 13411011006, 13411011009, 13411011010, 13668001901, 13668001905, 13668001930, 13668001974, 13668001990, 16590008630, 16590008656, 16590008660, 16590008690, 21695004615, 21695004630, 21695004645, 21695029615, 49999024915, 49999024930, 51079013501, 51079013520, 52959055030, 52959055060, 54569465900, 54569622600, 54868425300, 54868425301, 54868425302, 54868425303, 55045319606, 55045319608, 55045319609, 55111045405, 55111045430, 55111045490, 55289027730, 58016061500, 58016061530, 58016061560, 58016061590, 58864062930, 59762018101, 59762018102, 59762018103, 59762018104, 60505377903, 60505377909,

© 2024 Rodriguez MI et al. *JAMA Network Open.*

		63739051110, 64679071701, 64679071704, 64679071705, 65862052801, 65862052830, 65862052890, 65862052899, 66105011201, 66105011203, 66105011206, 66105011210, 66105011215, 67544019030, 67801041103, 68071032230, 68071032240, 68071032290, 68084048501, 68084048511, 68084070901, 68084070911, 68382003506, 68382003510, 68382003516, 93738201, 247229900, 247229990, 378488401, 490011400, 490011430, 490011460, 490011490, 603615016, 603615021, 603615025, 13811067510, 16714031401, 23155024901, 23155024909, 23490653006, 33358012530, 33358012560, 33358012590, 42291089550, 42291089590, 43063063390, 43353038230, 43353042130, 50268080115, 51079048201, 51079048220, 51079048230, 51079048256, 52959089030, 52959089060, 52959089090, 54569587200, 54868575400, 54868575401, 54868575402, 55111054805, 55111054890, 55887012560, 57237017501, 57237017590, 57664039588, 59762348302, 62332001131, 65162030709, 65862040701, 65862040730, 68001016000, 68084033101, 68084033111, 68084085601, 68084085611, 68382002101, 68382002110, 131326632, 131326646, 41616075981, 41616075983, 65580030203, 65580030209, 93474001, 93474010, 93474019, 93474093, 185037101, 247213200, 247213207, 247213230, 247213260, 247213290, 378623101, 378623105, 456401001, 591317601, 591317605, 781515701, 904608461, 10768706003, 12280006500, 13107000501, 13107000505, 13668000901, 13668000905, 13668000909, 13668000930, 13668000974, 16590005530, 16590005560, 16590005590, 18837024230, 21695003000, 21695065530, 23490755801, 23490755802, 23490755803, 24658014001, 24658014010, 24658014030, 31722020601, 31722020605, 35356002830, 35356004530, 42806001901, 42806001910, 43063048105, 45802030665, 45802030678, 54458098110, 54569632200, 54868498500, 54868498501, 54868527500, 54868527502, 55045342301, 55045342302, 55111034201, 55111034205, 55111034230, 55887026090, 55887050260, 57664050713, 57664050788, 58016003000, 58016003030, 58016003060, 58016003090, 58016059300, 58016059330, 58016059360, 58016059390, 58864084930, 59762480001, 59762480003, 59762480005, 59762480006, 60429017301, 60429017310, 60429017330, 60505251801, 60505251803, 60505251804, 60505251808, 63874114606, 65162005203, 65162005210, 65162005250, 65862000501, 65862000505, 65862000530, 68084058001, 68084058011, 68084073701, 69097082207, 69097082212, 76282020601, 76282020605, 76282020610, 54006258, 456413008, 17856054030, 54838054070, 60258083008, 65862007424, 93474101, 93474105, 93474119, 93474150, 93474193,

© 2024 Rodriguez MI et al. *JAMA Network Open.*

		185037201, 247189300, 247189307, 247189330, 247189360, 247189377, 247189390, 247213300, 247213307, 247213330, 247213360, 247213390, 378623201, 378623205, 456402001, 456402063, 591317701, 591317705, 904608561, 10768719103, 13107000601, 13107000605, 13668001001, 13668001005, 13668001006, 13668001030, 13668001074, 16590005630, 16590005660, 16590005690, 21695003100, 21695003130, 23490722501, 23490722502, 23490722503, 23490722504, 23490722505, 23490722509, 24658014101, 24658014110, 24658014130, 31722020701, 31722020705, 31722020710, 33358008360, 42806002001, 42806002010, 43063006314, 43063006330, 43063006360, 43063006390, 45802059665, 45802059678, 49999069030, 49999078930, 49999078960, 49999078990, 52959001000, 52959001030, 52959001060, 52959077330, 52959077352, 52959077360, 54458092205, 54458098010, 54569470301, 54569562600, 54569562601, 54569562602, 54868415900, 54868415902, 54868415903, 54868415904, 54868517800, 54868517801, 54868517803, 55045270609, 55045334106, 55045334108, 55111034301, 55111034305, 55111034330, 55289082730, 55289088314, 55289088330, 55289088390, 55887026101, 55887026190, 57664050813, 57664050818, 57664050888, 58016017800, 58016017802, 58016017804, 58016017805, 58016017810, 58016017812, 58016017814, 58016017815, 58016017820, 58016017830, 58016017840, 58016017850, 58016017860, 58016017889, 58016017890, 58016017899, 58016059800, 58016059830, 58016059860, 58016059890, 58864085230, 59762480101, 59762480103, 59762480105, 59762480106, 60429017401, 60429017410, 60429017430, 60505251901, 60505251903, 60505251904, 60505251908, 63629131801, 63874060901, 63874060910, 63874060920, 63874060930, 63874060960, 63874060990, 63874111303, 63874111306, 64720017111, 65162005303, 65162005310, 65162005350, 65862000601, 65862000605, 65862000630, 66336099330, 68071023190, 68084006601, 68084074401, 69097082307, 69097082312, 76282020701, 76282020705, 76282020710, 76282020730, 93474201, 93474205, 93474219, 93474250, 93474293, 185037301, 185037310, 247189407, 247189430, 247189460, 247189477, 247189490, 247213400, 247213407, 247213430, 247213460, 247213490, 378623301, 378623305, 456404001, 591317801, 591317805, 904608661, 10768761603, 13107000701, 13107000705, 13668001101, 13668001105, 13668001108, 13668001130, 13668001174, 21695003200, 21695003230, 23490722603, 23490722605, 23490722606, 23490722609, 24658014201, 24658014210, 24658014230, 31722020801, 31722020805, 31722020830, 35356004630,

© 2024 Rodriguez MI et al. *JAMA Network Open.*

		42806002101, 42806002110, 43063038501, 43063038530, 43063038590, 43063067030, 43063067090, 45802061665, 45802061678, 49999065400, 49999065430, 52959098630, 54569487900, 54569562700, 54569562701, 54868123900, 54868123901, 54868123902, 54868123903, 54868123904, 54868422600, 54868422601, 55045270500, 55045342400, 55045342408, 55111034401, 55111034405, 55111034430, 55887023730, 55887023760, 55887023786, 55887023790, 57664050913, 57664050918, 57664050988, 58016022400, 58016022402, 58016022404, 58016022405, 58016022410, 58016022412, 58016022414, 58016022415, 58016022420, 58016022430, 58016022440, 58016022450, 58016022460, 58016022489, 58016022490, 58016022499, 58864061615, 58864061630, 58864081215, 59762480201, 59762480203, 59762480205, 59762480206, 60429017501, 60429017510, 60429017530, 60505252001, 60505252003, 60505252004, 60505252008, 63874115703, 64720017211, 65162005403, 65162005410, 65162005450, 65243031003, 65243031903, 65862000701, 65862000705, 65862000730, 66336027130, 67544036015, 67544036615, 67544040915, 67544090615, 67544090673, 68071022230, 68645025254, 68645028254, 68645046754, 69097082407, 69097082412, 76282020801, 76282020805, 76282020810, 76282020830, 93585101, 93585105, 93585193, 143980801, 143980805, 247211730, 378385610, 378385677, 378385693, 456201001, 456201011, 456201063, 904631461, 904642661, 13668013601, 13668013605, 13668013610, 16590013930, 16590013960, 16729016901, 16729016917, 18837007690, 21695007315, 21695007330, 21695007345, 31722025090, 33342003711, 43063037401, 43063037430, 43063037490, 43063038930, 43063066601, 43063066630, 43063066690, 43547028110, 49999060000, 49999060015, 49999060030, 51079054301, 51079054320, 51079054356, 52959070300, 52959070330, 52959070360, 54569548300, 54569630400, 54868470000, 54868470001, 54868470002, 54868470003, 54868470004, 54868470005, 54868470006, 54879001001, 55045293400, 55045293408, 55289076830, 58016097700, 58016097702, 58016097730, 58016097760, 58016097790, 59746028001, 60505278101, 60505278108, 63874100901, 63874100903, 63874100906, 63874100909, 65862037401, 65862037405, 66105096903, 67801040930, 68001019600, 68001019603, 68084061701, 68084061711, 68180013501, 68387012730, 68645044870, 69097084805, 76282025010, 76282025090, 93585201, 93585205, 93585293, 143980701, 143980705, 247226230, 378385710, 378385777, 378385793, 456202001, 456202011, 456202063, 904631561, 904642761, 13668013701, 13668013705, 13668013710,

© 2024 Rodriguez MI et al. *JAMA Network Open.*

		16590049730, 16590049760, 16590049772, 16590049790, 16729017001, 16729017017, 21695007415, 21695007430, 31722025190, 33342003811, 33358020930, 43063039030, 43063052130, 43063052190, 43063066130, 43063066190, 43547028210, 43547028211, 49999062700, 49999062730, 49999062760, 51079054401, 51079054420, 51079054456, 52959070400, 52959070430, 52959070460, 54569548400, 54569630500, 54868477500, 54868477501, 54868477502, 54868477503, 55045298500, 55045298508, 55289082830, 59746028101, 60505278201, 60505278208, 63629298101, 65862037501, 65862037505, 66105097703, 67801031030, 68001019700, 68001019703, 68084061801, 68084061811, 68180013601, 68387012830, 68645044770, 69097084905, 76282025110, 76282025190, 93585001, 93585005, 93585093, 143980901, 378385577, 378385593, 456200501, 13668013501, 13668013505, 13668013510, 16729016801, 16729016817, 31722024990, 33342003611, 33358020830, 36818013701, 54868595100, 58016002700, 58016002730, 58016002760, 58016002790, 59746027901, 60505278001, 60505278008, 65862037301, 65862037305, 68001019500, 68180013701, 69097084705, 76282024990, 456210108, 591415138, 31722056924, 51672134801, 54838055170, 65162070588, 65862024824, 93104201, 93104210, 93104219, 93104293, 93722519, 93722528, 247196500, 247196503, 247196514, 247196530, 247196560, 247196590, 247196599, 378421001, 378541028, 406066101, 406066105, 406066191, 555087602, 777310402, 781282301, 781282310, 904578461, 13411017201, 13411017203, 13411017206, 13411017209, 13411017210, 16590009930, 16590009960, 16714035101, 16714035102, 16714035103, 21695005220, 21695005230, 21695005260, 21695005290, 23490560103, 33358014430, 33358014460, 33358014490, 42543072501, 42543072510, 43063057028, 43063057030, 43063057090, 50111064701, 50111064702, 50111064703, 50268033315, 51079099701, 51079099720, 52959066530, 52959066930, 52959066959, 52959066960, 54569412900, 54569531900, 54569531901, 54569531902, 54868303302, 54868566300, 54868566301, 54868566302, 55045290700, 55045290702, 55045290706, 55045290708, 55045290709, 55045376608, 55111014701, 55289030807, 55289030814, 55289030830, 55289061314, 55289061330, 55289061360, 55887045860, 55887045890, 60429071818, 60429071830, 60429071860, 60429071890, 61392023430, 61392023451, 61392023454, 61392023456, 61392023460, 61392023490, 61392023491, 62332002231, 63304068630, 63304068690, 63629160901, 63629160902, 63874057401, 63874057404, 63874057410, 63874057414, 63874057420,

© 2024 Rodriguez MI et al. *JAMA Network Open.*

		63874057421, 63874057428, 63874057430, 63874057460, 63874057472, 63874057474, 63874057477, 63874057490, 65862019201, 65862019205, 65862019299, 66267057630, 66336084430, 68115014390, 68387012530, 68645013154, 93718810, 93718856, 378073401, 378073493, 430021014, 555020101, 21695032000, 21695032090, 49884073401, 49884073410, 49884073411, 49999036214, 49999036230, 52959099130, 54868456000, 54868456001, 63874105301, 63874105302, 63874105303, 63874105304, 63874105305, 63874105306, 63874105308, 63874105309, 93435601, 93435605, 93435610, 93435619, 93435693, 93722619, 93722628, 185008510, 247037204, 247037210, 247191503, 247191507, 247191530, 247191590, 378422001, 378542028, 406066301, 406066303, 406066305, 406066362, 406066391, 555087702, 555087704, 555087705, 555087707, 777310502, 777310507, 777310530, 777310533, 781282201, 781282210, 904578561, 10544033630, 13411017301, 13411017303, 13411017306, 13411017309, 13411017310, 16590010030, 16590010060, 16590010090, 16714035201, 16714035202, 16714035203, 21695005330, 21695005360, 21695005390, 23490560201, 23490560202, 23490560203, 23490924006, 33358014530, 33358014560, 42543072601, 42543072610, 43063000901, 49999012800, 49999012814, 49999012820, 49999012830, 49999012860, 49999012890, 50111064801, 50111064803, 50268033415, 51079097101, 51079097119, 51079097120, 52959023300, 52959023310, 52959023314, 52959023320, 52959023330, 52959023340, 52959023350, 52959073200, 52959073210, 52959073214, 52959073215, 52959073220, 52959073230, 52959073240, 52959073250, 52959073260, 52959073290, 54569173202, 54569173203, 54569173204, 54569173205, 54569173206, 54569529100, 54569529101, 54569529103, 54569801300, 54569852200, 54569852201, 54868453700, 54868453701, 54868453702, 54868453703, 55045290800, 55045290802, 55045290806, 55045290808, 55045290809, 55111014801, 55111014810, 55289021522, 55289021530, 55289061028, 55289061030, 55289061060, 55289061090, 55887066160, 55887066190, 57866092201, 57866092202, 57866092203, 58016082810, 58016082820, 58016082830, 58016082840, 58016082860, 58016082890, 58016090500, 58016090502, 58016090503, 58016090510, 58016090520, 58016090530, 58016090560, 58016090573, 58016090589, 58864010315, 58864010330, 58864010360, 58864091730, 60429071918, 60429071930, 60429071960, 60429071990, 60760064730, 61392023515, 61392023530, 61392023545, 61392023551, 61392023554, 61392023556, 61392023560, 61392023590, 61392023591, 62037053905, 62037053910, 62332002331,

		63304068730, 63304068790, 63629161001, 63629161002, 63629161003, 63629161004, 63739029610, 63874057301, 63874057304, 63874057310, 63874057314, 63874057315, 63874057320, 63874057321, 63874057328, 63874057330, 63874057360, 63874057372, 63874057374, 63874057377, 63874057390, 65862019301, 65862019305, 65862019399, 66105056403, 66267048860, 66267048890, 66336000430, 66336000460, 67544029530, 67544052830, 67544084830, 68084060501, 68084060511, 68387012030, 68387012060, 68387012090, 68645013054, 378073501, 378073593, 430022014, 16241075901, 21695032100, 21695032130, 21695032160, 21695032190, 21695059630, 49884073501, 49884073510, 49884073511, 57866092001, 57866092002, 57866092003, 63874082801, 63874082804, 63874082814, 63874082820, 63874082821, 63874082828, 63874082830, 63874082860, 63874082890, 93610812, 121072104, 121472105, 49884069937, 54838052340, 60432016204, 93434656, 93719801, 93719805, 93719819, 93719856, 93719893, 247196430, 247196490, 378435093, 777310730, 781282401, 781282410, 781282431, 16590049030, 16590049060, 16590049072, 16590049090, 16714035301, 16714035302, 16714035303, 16714035304, 21695005430, 21695005460, 21695005490, 23490732703, 42543072701, 42543072705, 42543072730, 43063019730, 43063019790, 49884074301, 49884074305, 49884074311, 49884087201, 49884087205, 49884087211, 49999088630, 50268033515, 52959071730, 54569532001, 54868439400, 54868456200, 55045313708, 55111014901, 55111014930, 58016070400, 58016070402, 58016070403, 58016070430, 58016070460, 58016070473, 58016070489, 58016070490, 62332002431, 62332002491, 63304063201, 63304063230, 63304063290, 63874108003, 63874108006, 65862019401, 65862019405, 65862019430, 65862019499, 66267058730, 68001012900, 68001012903, 68001012904, 68084010101, 68258700303, 49909000530, 2300475, 555087154, 555087188, 52959063804, 55111028448, 228284803, 10370017511, 68727060001, 93005701, 185015701, 185015705, 378041401, 555096902, 35356003130, 42769122100, 51079099301, 51079099320, 57664036213, 57664036288, 60429076001, 60429076030, 60429076090, 60505016601, 62559016001, 63672110002, 228284903, 10370017611, 68727060101, 93007201, 185001701, 378040701, 555096702, 35356002930, 42769122200, 57664035788, 60429075801, 60505016401, 62559015801, 185002701, 185002705, 378041201, 555096802, 35356003030, 42769122500, 51079099201, 51079099220, 57664036113, 57664036188, 60429075901, 60429075930, 60429075990, 60505016501, 62559015901, 68084083721, 68084083725, 68084083795,

© 2024 Rodriguez MI et al. *JAMA Network Open.*

		29321013, 93711498, 247181408, 247181414, 247181430, 247208530, 378700110, 378700193, 406209703, 406209705, 406209790, 904567661, 904610961, 13107015405, 13107015430, 13107015490, 16590032230, 16590032256, 16590032260, 16590032272, 16590032290, 21695010130, 21695010160, 21695010190, 23490694701, 23490694702, 33261009010, 33261009014, 33261009020, 33261009028, 33261009030, 33261009060, 33261009090, 43547034703, 43547034709, 43547034711, 49884087605, 49999059730, 49999063130, 49999063160, 50268064015, 52959063930, 52959077530, 52959077550, 52959077560, 54458099010, 54569478700, 54569631800, 54868406500, 54868508000, 55045317108, 55289003745, 55289003790, 55887043330, 57664042113, 57664042183, 57664042199, 58016066100, 58016066130, 58016066160, 58016066190, 58016081700, 58016081702, 58016081703, 58016081730, 58016081760, 58016081790, 59762180801, 59762180802, 59762180803, 60429073405, 60429073430, 60429073490, 60505009701, 60505009702, 60505009704, 60505366303, 62037084530, 63629320501, 63739040710, 63739088810, 63874076710, 63874076714, 63874076730, 63874112506, 65862015430, 66105011603, 67544036515, 68084004401, 68084004411, 68084004485, 68115091930, 68382009701, 68382009705, 68382009706, 68382009710, 68382009716, 29321548, 60505037401, 60505040205, 29460613, 378200305, 378200393, 16590051430, 16590051460, 16590051472, 16590051490, 21695015915, 21695015930, 49999078030, 51079082463, 54868534700, 55887049030, 55887049060, 55887049082, 55887049090, 58016089700, 58016089730, 58016089760, 58016089790, 60505366803, 60505367303, 29321113, 29321159, 93711598, 247078707, 247078710, 247078714, 247078715, 247078728, 247078730, 247078750, 247078760, 247205200, 247205207, 247205230, 247205290, 378700210, 378700293, 406209801, 406209803, 406209805, 406209890, 904567761, 904611061, 13107015505, 13107015530, 13107015590, 13107015599, 16590018130, 16590018160, 16590018190, 18837012130, 21695010230, 21695010260, 21695010290, 21695046590, 23490605901, 23490605902, 23490605903, 33261009110, 33261009114, 33261009121, 33261009128, 33261009130, 33261009140, 33261009160, 33261009190, 33358028330, 33358028360, 33358028390, 43063017090, 43547034803, 43547034809, 43547034811, 49884087701, 49884087705, 49999063200, 49999063230, 49999063260, 50268064115, 51079077401, 51079077420, 52959036012, 52959036015, 52959036020, 52959036030, 52959036060, 52959077630, 52959077660, 54458098910, 54569381000, 54569554100, 54569554101,

© 2024 Rodriguez MI et al. *JAMA Network Open.*

		54569554102, 54569860900, 54868297600, 54868297602, 54868297603, 54868493700, 54868493701, 54868493702, 54868493703, 55045254608, 55045317208, 55045317209, 55154667304, 55154667307, 55289021630, 55289097230, 55289097260, 55289097290, 55887039490, 55887054990, 57664042213, 57664042218, 57664042283, 57664042299, 58016048500, 58016048502, 58016048510, 58016048512, 58016048515, 58016048520, 58016048525, 58016048530, 58016048540, 58016048550, 58016048560, 58016048570, 58016048580, 58016048590, 58016081800, 58016081802, 58016081803, 58016081830, 58016081860, 58016081890, 58016081899, 58864037230, 58864074115, 58864074130, 59762181001, 59762181002, 59762181003, 59762181004, 60429073501, 60429073505, 60429073530, 60429073590, 60505008300, 60505008301, 60505008302, 60505008304, 60505366403, 62037084601, 62037084610, 62037084630, 63629184001, 63739096310, 63874053801, 63874053810, 63874053814, 63874053815, 63874053820, 63874053830, 63874053860, 65862015505, 65862015530, 65862015599, 66105011810, 66336079930, 67544031715, 67544045515, 68084004501, 68084004511, 68084004585, 68382009801, 68382009805, 68382009806, 68382009810, 68382009816, 29460713, 378200405, 378200493, 21695016030, 49999060115, 51079082562, 51079082563, 52959079230, 54569559800, 54868479100, 55887051120, 55887051130, 55887051160, 55887051182, 55887051190, 58016076100, 58016076130, 58016076160, 58016076190, 60505366903, 60505367403, 29321213, 93711698, 247121403, 247121407, 247121430, 247209430, 247209490, 378700310, 378700393, 406209903, 406209905, 406209990, 904567861, 904611161, 13107015605, 13107015630, 13107015690, 13107015699, 16590051206, 16590051230, 16590051260, 16590051272, 16590051290, 21695010330, 21695010390, 23490606001, 23490606002, 43547034909, 49884087805, 49999061330, 50268064215, 54458098810, 54868352600, 54868493800, 57664042413, 57664042483, 57664042499, 58016074900, 58016074902, 58016074930, 58016074960, 58016074990, 58016080600, 58016080630, 58016080660, 58016080690, 59762181201, 59762181202, 59762181203, 60429073605, 60429073630, 60429073690, 60505008401, 60505008402, 60505008404, 60505366503, 63874100303, 63874115103, 65862015630, 65862015699, 66105011703, 67544050015, 68084004601, 68084004611, 68084004685, 68382009901, 68382009905, 68382009906, 68382009910, 68382009916, 29320813, 378200593, 378200693, 54868536500, 58016090700, 58016090730, 58016090760, 58016090790, 60505367003, 60505367503, 29321313, 93712198, 247226530,

© 2024 Rodriguez MI et al. *JAMA Network Open.*

		247226560, 247226590, 378700410, 378700493, 406200103, 406200105, 406200190, 904611261, 13107015705, 13107015730, 13107015790, 13107015799, 16590051330, 16590051356, 16590051360, 16590051372, 16590051390, 21695010430, 21695010490, 23490694801, 23490694802, 23490694803, 43353042915, 43547035003, 43547035009, 43547035011, 49884087905, 49999082830, 50268064315, 51079077501, 51079077520, 54569568200, 54569568201, 54868396200, 54868481700, 54868481701, 55289005345, 55289005390, 57664042513, 57664042583, 57664042599, 58016073100, 58016073130, 58016073160, 58016073190, 58864062815, 58864071630, 59762181501, 59762181502, 59762181503, 60429073705, 60429073715, 60429073730, 60429073745, 60429073790, 60505010101, 60505010102, 60505010104, 60505010107, 60505366603, 63739040810, 63874067010, 63874067015, 63874067020, 63874067030, 65862015730, 65862015799, 67544043415, 68084004701, 68084004711, 68084004785, 68115091630, 68382000101, 68382000105, 68382000106, 68382000110, 68382000116, 68382010006, 68968907503, 68968201001, 68968202001, 68968203001, 68968204001, 49491030, 49491041, 143958005, 143958009, 143958030, 143965405, 143965409, 143965430, 228272309, 228272396, 247037103, 247037145, 247227200, 247227230, 247227260, 247227290, 378418801, 378418805, 378418893, 378812701, 378812705, 904608961, 904633361, 13411015301, 13411015303, 13411015306, 13411015309, 13411015315, 13668000601, 13668000605, 13668000610, 13668000630, 13668000650, 13668000690, 16252053530, 16252053550, 16252053590, 16590025130, 16590025160, 16590025190, 16590041630, 16714061301, 16714061304, 16714061305, 16714061306, 18837018560, 18837018590, 21695016630, 21695016690, 23490626301, 23490626302, 23490626303, 31722021405, 31722021430, 31722021490, 33358032330, 43063041390, 43353036215, 43353036415, 43353041530, 43353041915, 43353044715, 49999037500, 49999037515, 49999037530, 49999086130, 49999086160, 49999086190, 51079015101, 51079015120, 51079076401, 51079076420, 52959078130, 52959078160, 52959087630, 52959087660, 54458094510, 54569357500, 54569357501, 54569581900, 54569581901, 54569581902, 54868263707, 54868263708, 54868563800, 54868563801, 54868563802, 54868563805, 54868563806, 55045220801, 55045220803, 55045220807, 55045220808, 55045356201, 55289038130, 55289038145, 55289038160, 55289038190, 55289055015, 55289055030, 55887092530, 58016000900, 58016000930, 58016000960, 58016000990, 58016066800, 58016066830, 58016066860,

© 2024 Rodriguez MI et al. *JAMA Network Open.*

		58016066890, 58864062715, 58864062730, 59762491001, 59762491002, 59762491003, 59762491004, 59762491005, 63304016601, 63304016605, 63304016630, 63629328901, 63874059601, 63874059610, 63874059614, 63874059615, 63874059620, 63874059630, 63874059660, 64679075301, 64679075304, 64679075307, 65243006303, 65862001301, 65862001305, 65862001330, 65862001390, 67544008015, 67544090815, 67801020510, 68084018201, 68084018211, 68115036545, 68115036560, 68180035301, 68180035302, 68180035305, 68180035306, 68180035309, 68645042654, 68645048970, 68645050001, 69097083502, 69097083512, 76282021401, 76282021405, 76282021490, 49494023, 16714060101, 16714060102, 59762494001, 63304084005, 49496030, 143958209, 143958230, 143965609, 143965630, 228272109, 228272150, 378418601, 378418605, 378418693, 378801101, 378801105, 904608761, 904633161, 13668000401, 13668000405, 13668000410, 13668000430, 13668000450, 13668000490, 16252053330, 16252053350, 16590024930, 16590024960, 16590024990, 16714061101, 16714061104, 16714061105, 16714061106, 21695016430, 23490705001, 23490705002, 31722021205, 31722021230, 31722021290, 33358032130, 35356003330, 49999077630, 49999077650, 51079014901, 51079014920, 51079076201, 51079076220, 52959078730, 52959087230, 52959087260, 54458094710, 54569452900, 54569452901, 54569452902, 54569619200, 54868565800, 55045338608, 55289037830, 55887016015, 55887016030, 55887016060, 55887016090, 58016001100, 58016001130, 58016001160, 58016001190, 58016066400, 58016066430, 58016066460, 58016066490, 59762496001, 63304016430, 63629331301, 65862001101, 65862001105, 65862001130, 65862001190, 68084018001, 68084018011, 68115076530, 68180035106, 68180035109, 68645042454, 68645048770, 68645049801, 69097083302, 69097083312, 76282021201, 76282021205, 76282021290, 49490030, 49490041, 143958105, 143958109, 143958130, 143965505, 143965509, 143965530, 228272209, 228272290, 247067503, 247067508, 247067533, 247227130, 378418701, 378418705, 378418793, 378812101, 378812105, 904608861, 904633261, 13411015201, 13411015203, 13411015206, 13411015209, 13411015215, 13668000501, 13668000505, 13668000510, 13668000530, 13668000550, 13668000590, 16252053430, 16252053450, 16252053490, 16590025030, 16590025060, 16590025090, 16714061201, 16714061204, 16714061205, 16714061206, 21695016530, 23490626401, 23490626402, 23490626403, 23490626404, 31722021305, 31722021330, 31722021390, 33358032230, 43063003301, 43063041160, 43063041190, 49999029215,

© 2024 Rodriguez MI et al. *JAMA Network Open.*

		49999029230, 49999086030, 49999086060, 51079015001, 51079015020, 51079076301, 51079076320, 51655066224, 52959036100, 52959036114, 52959036130, 52959036160, 52959087530, 52959087560, 52959087590, 54458091302, 54458092405, 54458094405, 54458094410, 54569372400, 54569372401, 54569372403, 54569372404, 54569372405, 54569581800, 54569581801, 54569581802, 54569857900, 54868563900, 54868563901, 54868563902, 54868563903, 55045222400, 55045222402, 55045222407, 55045222408, 55045356601, 55289029114, 55289029130, 55289029160, 55289040930, 55289040960, 55887016830, 55887016860, 55887016890, 58016001000, 58016001030, 58016001060, 58016001090, 58016036600, 58016036630, 58016036660, 58016036690, 58864070730, 59762490001, 59762490002, 59762490003, 59762490004, 59762490005, 63304016501, 63304016505, 63304016530, 63629330901, 63874055501, 63874055510, 63874055514, 63874055515, 63874055520, 63874055530, 63874055560, 64679075201, 64679075204, 64679075207, 65862001201, 65862001205, 65862001230, 65862001290, 67544029815, 67801020410, 68084018101, 68084018111, 68115036660, 68180035201, 68180035202, 68180035205, 68180035206, 68180035209, 68645042554, 68645048870, 68645049901, 69097083402, 69097083412, 76282021301, 76282021305, 76282021330, 76282021390, 378006001, 378008701, 378009201, 52010530, 52010590, 93720619, 93720656, 93720693, 185002010, 185002030, 247184303, 247184307, 247184308, 247184314, 247208330, 247208360, 247208390, 378351501, 378351510, 378351570, 378351593, 591111710, 591111730, 904651961, 13107003105, 13107003130, 13107003134, 16590015330, 16590015360, 18837009360, 21695008100, 21695008130, 21695008160, 23490698901, 23490698902, 23490922000, 23490922003, 49999062930, 51079008601, 51079008620, 51079008630, 51079008656, 52959077430, 52959077460, 52959090130, 54569589500, 54868484800, 57237000805, 57237000830, 57664049918, 57664049983, 58016062400, 58016062430, 58016062460, 58016062490, 58016089400, 58016089402, 58016089403, 58016089430, 58016089460, 58016089490, 58016089499, 59762141603, 59762141605, 59762141606, 59762141609, 60505024701, 60505024708, 62037075310, 62037075330, 62584055601, 62584055611, 62584055685, 63739035504, 63739035510, 63874109803, 65862003101, 65862003103, 65862003105, 65862003130, 65862003190, 67544042915, 68084011901, 68084011911, 68387036060, 52010606, 52010630, 52010690, 93730318, 93730365, 591223015, 591246915, 23490921000, 23490921003, 57237001106, 58016482501,

© 2024 Rodriguez MI et al. *JAMA Network Open.*

		59762141007, 65862002106, 66993070930, 52010730, 52010790, 93720719, 93720756, 93720793, 185021210, 185021230, 247191815, 247191830, 247208430, 247208460, 378353001, 378353005, 378353070, 378353093, 591111810, 591111830, 13107000305, 13107000330, 13107000334, 16590015430, 16590015460, 21695008200, 21695008230, 21695008260, 23490699003, 43353037845, 43353053645, 49999063030, 51079008701, 51079008720, 51079008730, 51079008756, 54569596800, 54569596802, 54868544800, 57237000905, 57237000930, 57664050018, 57664050083, 57866306102, 58016072300, 58016072330, 58016072360, 58016072390, 58016098600, 58016098602, 58016098603, 58016098630, 58016098660, 58016098690, 58864082315, 58864082330, 59762141703, 59762141705, 59762141706, 59762141709, 60505024801, 60505024808, 62037075410, 62037075430, 62584055701, 62584055711, 63739035610, 63874102003, 63874109703, 65862000301, 65862000303, 65862000305, 65862000330, 65862000390, 66105055003, 67544016915, 67544043015, 68084012001, 68084012011, 68258700503, 68387036307, 68387036315, 68387036330, 52010806, 52010830, 52010890, 93730418, 93730465, 591223115, 591247015, 57237001206, 58016482601, 59762141207, 65862002206, 66993071130, 52010930, 93720819, 93720856, 93720893, 185022210, 185022230, 378354501, 378354505, 378354570, 378354593, 591111930, 13107003205, 13107003234, 16590015530, 16590015560, 21695008330, 23490729303, 35356003230, 51079008801, 51079008820, 51079008830, 51079008856, 54868581200, 57237001005, 57237001030, 57664050118, 57664050183, 58016028200, 58016028230, 58016028260, 58016028290, 58016060000, 58016060030, 58016060060, 58016060090, 59762141803, 59762141805, 59762141806, 59762141809, 60505024901, 60505024908, 62037075510, 62037075530, 63874115503, 65862003201, 65862003203, 65862003205, 65862003230, 65862003290, 68084012101, 68084012111, 52011006, 52011030, 52011090, 93730502, 93730565, 591247115, 57237001306, 59762141407, 65862002306, 66993071230, 13107000105, 13107000130, 57237000705, 57237000730, 57664051018, 57664051083, 59762141503, 59762141505, 59762141506, 59762141509, 65862000101, 65862000105, 65862000130, 378261001, 378261010, 440709030, 603221202, 603221216, 603221221, 603221232, 781148601, 781148610, 16590001130, 16590001156, 16590001160, 16590001172, 16590001190, 16714044601, 16714044602, 16729017101, 16729017117, 21695044630, 23490504700, 23490504701, 23490504702, 23490504703, 23490504704, 49727015902, 49727015905,

© 2024 Rodriguez MI et al. *JAMA Network Open.*

		49999020500, 49999020530, 49999020560, 51079013101, 51079013120, 51079013163, 52959000802, 52959000815, 52959000830, 52959000840, 52959000860, 52959000890, 54569017200, 54569017201, 54569017202, 54569017204, 54569017206, 54569017208, 54868006402, 54868006403, 54868006404, 54868006405, 54868006406, 54868006407, 55045168200, 55045168201, 55045168202, 55045168203, 55045168204, 55045168205, 55045168206, 55045168208, 55045168209, 55289012412, 55289012430, 55887057214, 55887057290, 57866307101, 58016081300, 58016081302, 58016081303, 58016081310, 58016081312, 58016081315, 58016081320, 58016081324, 58016081330, 58016081350, 58016081360, 58016081373, 58016081389, 58016081390, 58016081399, 58864002430, 60760021215, 60760021230, 61392014334, 61392014345, 61392014356, 61392014391, 63874031101, 63874031102, 63874031104, 63874031110, 63874031112, 63874031115, 63874031120, 63874031124, 63874031130, 63874031140, 63874031150, 63874031160, 63874031172, 63874031174, 63874031177, 63874031182, 63874031190, 66267001860, 66267001890, 66336035430, 66336035460, 66336035490, 67544020630, 68115002220, 68387033630, 68387033690, 378268501, 378268593, 603221621, 603221625, 781149001, 16590043730, 16714045001, 16729017501, 21695025330, 43063019360, 49999031800, 49999031830, 49999031860, 51079056301, 51079056320, 52959054214, 52959054215, 52959054221, 52959054228, 52959054230, 52959054240, 52959054242, 54569214601, 54569214602, 54868243300, 54868243302, 55045159200, 55045159201, 55045159205, 55045159206, 55045159208, 55045159209, 58016085800, 58016085814, 58016085821, 58016085828, 58016085830, 58016085840, 58016085850, 58016085860, 58016085899, 58864075730, 61392015331, 61392015332, 61392015339, 61392015345, 61392015354, 61392015356, 61392015391, 63874115803, 66267056090, 66336022430, 67046001530, 68115002160, 68387033930, 378269501, 378269593, 603221721, 781149101, 16714045101, 16729017601, 51079056401, 51079056420, 54868243400, 54868243402, 57866390701, 58016071000, 58016071030, 58016071060, 58016071090, 58016071099, 378262501, 378262510, 440709130, 603221321, 603221330, 603221332, 781148701, 781148710, 904020161, 10544032930, 16590001215, 16590001230, 16590001260, 16590001290, 16714044701, 16714044702, 16729017201, 16729017217, 21695025130, 21695025160, 21695025190, 23490505000, 23490505001, 23490505004, 23490505005, 23490505006, 23490505008, 23490505009, 33358002330, 49999006300, 49999006315, 49999006330,

© 2024 Rodriguez MI et al. *JAMA Network Open.*

		49999006350, 49999006360, 49999006390, 51079010701, 51079010717, 51079010719, 51079010720, 51079010763, 52959034805, 52959034810, 52959034812, 52959034814, 52959034815, 52959034820, 52959034830, 52959034850, 52959034860, 52959034890, 54569017500, 54569017501, 54569017502, 54569017503, 54569017504, 54569017505, 54569017506, 54569017508, 54868006502, 54868006503, 54868006504, 54868006505, 54868006507, 54868006508, 54868006509, 55045146300, 55045146301, 55045146302, 55045146303, 55045146304, 55045146306, 55045146307, 55045146308, 55045146309, 55289073001, 55289073012, 55289073025, 55289073030, 55289073060, 55289073090, 55887057040, 55887057092, 57866307201, 57866307202, 57866307203, 58016081400, 58016081402, 58016081415, 58016081420, 58016081425, 58016081430, 58016081440, 58016081445, 58016081450, 58016081460, 58016081489, 58016081490, 58016081499, 58864002215, 58864002230, 60429001618, 60429001630, 60429001660, 60429001690, 61392014034, 61392014045, 61392014056, 61392014091, 63629136901, 63874043001, 63874043002, 63874043004, 63874043014, 63874043015, 63874043020, 63874043025, 63874043028, 63874043030, 63874043040, 63874043050, 63874043060, 63874043090, 65243000603, 66267001990, 66336002730, 66336002760, 67544008530, 67544008560, 68115002314, 68115002348, 68387033515, 68387033524, 68387033530, 68387033560, 68387033590, 69874042210, 378265001, 378265010, 440709230, 603221421, 603221432, 781148801, 781148810, 904020261, 16590001330, 16590001360, 16590001390, 16714044801, 16714044802, 16729017301, 16729017317, 23490505101, 23490505102, 23490505103, 33358002430, 49999022800, 49999022830, 49999022860, 49999022890, 51079013301, 51079013320, 51079013363, 52959051401, 52959051410, 52959051420, 52959051421, 52959051430, 52959051460, 52959051490, 54348051130, 54569151901, 54569151902, 54569151903, 54868006602, 54868006603, 54868006605, 54868006606, 55045174100, 55045174101, 55045174102, 55045174103, 55045174105, 55045174106, 55045174108, 55045174109, 55289001630, 55289001660, 57866307301, 57866307302, 57866307303, 58016081500, 58016081515, 58016081520, 58016081521, 58016081528, 58016081530, 58016081560, 58016081590, 58016081599, 58809071701, 58864002330, 60429001730, 61392014145, 61392014156, 61392014191, 63629136801, 63874035901, 63874035902, 63874035914, 63874035915, 63874035920, 63874035928, 63874035930, 63874035940, 63874035950, 63874035960, 63874035990, 66336067330, 66336067350, 66336067360,

© 2024 Rodriguez MI et al. *JAMA Network Open.*

		67544025330, 68115002490, 68387033724, 68387033730, 378267501, 378267593, 603221521, 781148901, 10544040530, 16714044901, 16729017401, 23490505203, 43063033630, 43063033660, 49999090900, 51079014701, 51079014720, 52959028400, 52959028430, 52959028460, 54569186401, 54569186402, 54868235700, 54868235702, 54868235703, 55045215301, 55045215308, 55887039860, 57866390601, 58016080800, 58016080830, 58016080860, 58016080890, 60760022130, 63874029601, 63874029604, 63874029615, 63874029620, 63874029630, 63874029640, 63874029660, 63874029690, 68387033830, 591571501, 591571630, 591571301, 591571401, 93095601, 378302501, 406880601, 406990601, 406990603, 406990662, 781202701, 51672401101, 51672401105, 51672401106, 60429028730, 68084079021, 68084079025, 68084079095, 93095801, 378305001, 406880701, 406990701, 406990703, 406990762, 781203701, 51672401201, 51672401205, 51672401206, 54868144700, 54868402300, 68084081825, 93096001, 378307501, 406880801, 406990801, 406990803, 781204701, 51672401301, 51672401305, 51672401306, 60429028830, 68000701, 247231400, 247231430, 247231460, 247231477, 247231490, 781197101, 781521801, 955103010, 23490539101, 45963034102, 52152034102, 52959012830, 52959012860, 54569200600, 55887014430, 55887014460, 55887014482, 55887014490, 55887014492, 58016050200, 58016050215, 58016050228, 58016050230, 58016050250, 58016050260, 69238105301, 69238105303, 68002001, 781197501, 955103410, 23490539201, 45963034502, 52152034502, 52152034504, 69238106101, 68002150, 781197650, 955103505, 45963034650, 52152034650, 69238106302, 68001101, 781197201, 955103110, 21695042800, 21695042828, 21695042830, 23490539301, 23490539302, 45963034202, 52152034202, 52152034205, 52959045814, 52959045820, 52959045830, 54569040400, 55045171802, 55045171806, 55045171808, 55045171809, 55887014330, 55887014360, 55887014382, 55887014390, 55887014392, 58016085300, 58016085302, 58016085303, 58016085314, 58016085315, 58016085320, 58016085328, 58016085330, 58016085350, 58016085360, 58016085373, 58016085389, 58016085390, 63874082901, 63874082910, 63874082914, 63874082915, 63874082920, 63874082928, 63874082930, 63874082942, 63874082950, 63874082960, 68084097225, 68084097295, 69238105501, 68001501, 591080901, 591080905, 781197301, 955103210, 16590048430, 16590048460, 16590048472, 16590048490, 23490539401, 23490539402, 23490539403, 45963034302, 52152034302, 52152034305, 52959046400, 52959046412, 52959046414, 52959046420, 54569170102, 63874076630, 63874076660,

© 2024 Rodriguez MI et al. *JAMA Network Open.*

		68115050930, 69238105701, 68001901, 781197401, 955103310, 45963034402, 52152034402, 69238105901, 247173130, 247173160, 247173190, 378104901, 378104910, 591562901, 21695044130, 33358011730, 33358011760, 51079043601, 51079043620, 51285091004, 52959053730, 52959053790, 54569246200, 54868231703, 54868231704, 55045183701, 55045183702, 55045183706, 55045183708, 55045183709, 58016066300, 58016066330, 58016066360, 58016066390, 61392072632, 61392072645, 61392072651, 61392072654, 61392072691, 62584068601, 62584068611, 63629322101, 93961212, 54838051240, 60432065104, 378641001, 378641010, 591563301, 641451386, 17236035911, 51079065101, 51079065120, 54868228406, 61392073030, 61392073031, 61392073032, 61392073039, 61392073045, 61392073051, 61392073060, 61392073090, 61392073091, 49884022201, 49884022203, 49884022205, 378312501, 378312510, 591211401, 23490548003, 33358011830, 33358011860, 51079043701, 51079043720, 51285091104, 52959054110, 52959054120, 52959054130, 52959054160, 54569217900, 54569217901, 54868006202, 54868006204, 55045188401, 55045188402, 55045188406, 55045188408, 55045188409, 55289037030, 55887035630, 55887035660, 58016083300, 58016083310, 58016083312, 58016083314, 58016083315, 58016083320, 58016083321, 58016083330, 58016083340, 58016083350, 58016083360, 58016083390, 61392072731, 61392072732, 61392072745, 61392072751, 61392072754, 61392072790, 61392072791, 62584068701, 62584068711, 63874057901, 63874057904, 63874057910, 63874057912, 63874057914, 63874057915, 63874057920, 63874057928, 63874057930, 63874057932, 63874057940, 63874057950, 63874057960, 63874057990, 66336055330, 68115012490, 378425001, 378425010, 591563101, 591563110, 16590008130, 16590008160, 16590008172, 16590008190, 33358011930, 33358011960, 49999019030, 51079043801, 51079043820, 51285091204, 52959066230, 54569169600, 54569169601, 54868196402, 54868196403, 55045208401, 55045208403, 55045208406, 55289001801, 55289001830, 55887028930, 55887028960, 58016083400, 58016083410, 58016083414, 58016083415, 58016083420, 58016083421, 58016083430, 58016083440, 58016083450, 58016083460, 58016083490, 61392072845, 61392072851, 61392072854, 61392072891, 62584068801, 62584068811, 63874063801, 63874063804, 63874063810, 63874063814, 63874063815, 63874063820, 63874063821, 63874063828, 63874063830, 63874063840, 63874063850, 63874063860, 63874063890, 66267068030, 378537501, 378537510, 440747830, 591563201, 17236035811, 51079064501, 51079064520, 54868255200,

© 2024 Rodriguez MI et al. *JAMA Network Open.*

		54868255202, 55289025830, 58016079700, 58016079730, 58016079760, 58016079790, 61392072930, 61392072932, 61392072945, 61392072951, 61392072954, 61392072960, 61392072991, 406992003, 781176201, 42291034501, 49884005401, 49884005410, 53489033001, 54569272600, 54738091201, 55045179408, 55045379901, 58016083900, 58016083902, 58016083912, 58016083915, 58016083920, 58016083930, 58016083960, 58016083990, 61392002545, 61392002554, 61392002556, 61392002591, 64125013301, 64125013310, 68115043930, 68180031101, 69315013301, 69315013310, 247168010, 406992103, 781176401, 781176410, 781176413, 16590057730, 42291034601, 49884005501, 49884005510, 49999040030, 51655014851, 51655014877, 52959079130, 53489033101, 54569019400, 54569019402, 54738091301, 54868134402, 54868134403, 54868134404, 55045172100, 55045172108, 55289014430, 55289014490, 57866393001, 58016084100, 58016084102, 58016084112, 58016084115, 58016084120, 58016084130, 58016084160, 58016084190, 61392002645, 61392002654, 61392002656, 61392002691, 62584075001, 62584075011, 64125013401, 64125013410, 68180031201, 69315013401, 69315013410, 247078907, 247078914, 247078930, 406992201, 406992203, 781176601, 781176610, 781176613, 42291034701, 49884005601, 49884005610, 51655022351, 53489033201, 54569019603, 54569019604, 54738091401, 54868222101, 54868222103, 54868222104, 54868222105, 55045172208, 55887028830, 57866393101, 58016086600, 58016086602, 58016086612, 58016086615, 58016086620, 58016086630, 58016086660, 58016086690, 61392002745, 61392002754, 61392002756, 61392002791, 63629151001, 64125013501, 64125013510, 68180031301, 69315013501, 69315013510, 54027413, 406992401, 406992403, 406993203, 68180031506, 54027513, 406992501, 406992503, 406993303, 68180031606, 54027613, 406992601, 406992603, 406993403, 68180031706, 54027313, 406992301, 406992303, 406993103, 52959090030, 68180031406, 93081001, 93081005, 247061610, 247061615, 247061630, 247105660, 406991001, 406991003, 591578601, 591578605, 21695009330, 21695009360, 21695009390, 23490601901, 23490601902, 23490601903, 23490601904, 33358027030, 33358027060, 33358027090, 49999053830, 51079080301, 51079080320, 51672400101, 51672400102, 51672400105, 51672400106, 52959035802, 52959035820, 52959035890, 54569022500, 54569414600, 54569414601, 54868283300, 54868283301, 54868283501, 54868283502, 54868283503, 55045192001, 55045192002, 55045192006, 55045192008, 55045192009, 55289058630, 55887043930, 55887043960,

		55887043992, 58016051900, 58016051902, 58016051912, 58016051915, 58016051920, 58016051930, 58016051940, 58016051950, 58016051960, 58016051970, 58016051980, 58016051990, 58016051999, 61392036145, 61392036156, 61392036191, 63629320401, 63874103301, 63874103303, 63874103304, 63874103306, 66336097360, 68084003101, 68084003111, 68115026145, 121067812, 121067816, 63304020201, 93081101, 93081105, 247105715, 247105760, 247107510, 247107530, 247107560, 406991101, 406991103, 591578701, 591578705, 591578710, 16590017130, 16590017160, 16590017190, 21695009430, 21695009460, 21695009490, 23490602001, 23490602002, 23490602003, 33358027101, 33358027130, 33358027160, 49999021525, 49999021530, 49999021560, 51079080401, 51079080419, 51079080420, 51672400201, 51672400202, 51672400205, 51672400206, 52959016330, 52959035950, 54569384900, 54569384901, 54569389400, 55045195601, 55045195602, 55045195604, 55045195606, 55045195608, 55045195609, 55289009930, 55289009950, 55887051815, 55887051830, 55887051890, 55887074550, 57866665001, 57866665002, 57866665003, 58016049100, 58016049102, 58016049115, 58016049120, 58016049125, 58016049130, 58016049140, 58016049150, 58016049160, 58016049170, 58016049180, 58016049190, 60760081160, 61392036445, 61392036456, 61392036491, 63629283302, 63629283304, 63739019010, 63874058001, 63874058014, 63874058015, 63874058020, 63874058030, 63874058060, 63874058090, 66336075760, 67544007830, 68084003201, 68084003211, 68387033015, 68387033030, 68387033060, 93081201, 93081205, 247167910, 247167960, 406991201, 406991203, 591578801, 591578805, 23490602106, 35356036960, 49999091590, 51079080501, 51079080520, 51672400301, 51672400302, 51672400305, 51672400306, 52959051915, 52959051930, 52959051960, 54868248101, 54868248102, 54868248103, 55045198202, 55045198208, 55045198209, 55887049830, 55887049860, 58016050800, 58016050815, 58016050830, 58016050860, 61392036745, 61392036756, 61392036791, 63874107906, 66336062160, 68084095832, 68084095833, 93081301, 93081305, 247037030, 247105860, 406991303, 591578901, 16590051030, 16590051060, 16590051072, 16590051090, 51672400401, 51672400402, 51672400405, 51672400406, 52959084060, 58016087500, 58016087512, 58016087515, 58016087520, 58016087530, 58016087560, 61392037045, 61392037054, 61392037056, 61392037091, 63874108101, 54021125, 555059402, 42806009701, 50111052403, 50383096010, 51285059402, 64980015901, 54021025, 555059502, 42806009601, 50111052303,

© 2024 Rodriguez MI et al. *JAMA Network Open.*

		50383095910, 51285059502, 64980015801, 45963029530, 51285055402, 45963029330, 51285053802, 52152029308, 52152029330, 45963029430, 51285053902, 52152029408, 52152029430, 42023012901, 55390046505, 60505066402, 63323027205, 59148001871, 59148004580, 59148001971, 59148007280, 65757040101, 65757040103, 65757040201, 65757040203, 65757040301, 65757040303, 703702101, 703702103, 703702301, 10147092205, 50458025414, 53150048505, 53150048905, 55390041301, 55390041305, 63323047101, 63323047105, 63323047151, 63323047155, 67457038158, 67457040913, 247235505, 703701101, 703701103, 703701301, 10147092103, 50458025303, 53150041510, 53150042205, 55390041201, 55390041205, 63323046901, 63323046905, 63323046951, 67457038258, 67457041013, 2763511, 2765801, 2763611, 2765901, 2763711, 2766001, 50458056201, 50458056301, 50458056401, 50458060601, 50458056001, 50458060701, 50458060801, 50458056101, 50458060901, 50458030911, 50458030611, 50458030711, 50458030811, 57844051011, 57844051055, 69011301, 69011302, 703704101, 703704103, 703704104, 703704501, 10147091101, 25021080601, 25021082310, 50458025501, 55390014701, 55390014710, 55390044701, 55390044710, 60505072703, 63323047401, 63323047410, 63323047491, 67457042612, 59148001665, 2759701, 517095501, 781315972, 781910572, 49392020, 49392083, 54838057059, 59148001315, 60505040405, 65162089374, 93758056, 904651161, 13668021805, 13668021830, 13668021890, 13811068110, 13811068130, 21695000315, 21695000330, 21695000345, 31722082730, 42291013530, 49999059815, 50268008915, 54868520200, 55289025130, 59148000813, 59148000835, 60505267403, 60687017901, 60687017911, 65162089803, 65162089809, 65862066305, 65862066330, 68115046915, 13811069230, 59148064023, 93758156, 904651204, 13668021905, 13668021930, 13668021990, 13811068210, 13811068230, 21695000415, 31722082830, 42291013630, 49999081730, 50268009012, 59148000913, 59148000935, 60505267503, 60687019111, 60687019121, 65162089903, 65162089909, 65862066405, 65862066430, 13811069330, 59148064123, 93761356, 904650904, 13668021630, 13668021690, 13811067910, 13811067930, 16590032330, 31722081930, 42291013330, 50268008712, 54868628000, 59148000613, 59148000635, 60505307503, 60687015711, 60687015721, 65162089603, 65162089609, 65862066105, 65862066130, 93758256, 904651304, 13668022005, 13668022030, 13668022090, 13811068310, 13811068330, 16590057330, 21695000530, 31722082930, 42291013730, 49999081830, 50268009112, 59148001013,

© 2024 Rodriguez MI et al. *JAMA Network Open.*

		59148001035, 60505267603, 60687020211, 60687020221, 65162090103, 65162090109, 65862066505, 65862066530, 93758356, 904651404, 13668022105, 13668022130, 13668022190, 13811068410, 13811068430, 31722083030, 35356017130, 42291013830, 50268009212, 55289056630, 59148001113, 59148001135, 60505267703, 60687021311, 60687021321, 65162090203, 65162090209, 65862066605, 65862066630, 93756956, 904651061, 12280028230, 13668021705, 13668021730, 13668021790, 13811068010, 13811068030, 16590074530, 16590074560, 21695000215, 21695000230, 31722082030, 42291013430, 49999081630, 50268008815, 58016005400, 58016005430, 58016005460, 58016005490, 59148000713, 59148000735, 60505267303, 60687016801, 60687016811, 65162089703, 65162089709, 65862066205, 65862066230, 59148003513, 59148003613, 59148003713, 59148003813, 59148003913, 59148004013, 61874011530, 61874017008, 61874013030, 61874014530, 61874016030, 78012705, 78012706, 78012761, 93436019, 93436060, 93436093, 93777201, 93777205, 93777293, 378086001, 378086005, 51079092201, 51079092220, 57664034713, 57664034788, 68084023401, 68084023411, 93301001, 93301019, 93301084, 378381501, 18860010401, 18860010410, 68322000202, 68322000204, 93301101, 18860010110, 64597010110, 93308601, 93308619, 93308684, 18860010501, 18860010510, 93440501, 93440505, 93440519, 93440593, 378097301, 378097305, 51079074901, 51079074920, 93308701, 93308719, 93308784, 18860010601, 18860010610, 78012605, 78012606, 78012661, 93435901, 93435905, 93435919, 93435960, 93435993, 378082501, 51079092101, 51079092120, 57664034513, 57664034588, 68084023301, 68084023311, 93301201, 93301219, 93301284, 378381301, 18860010201, 18860010210, 68322000102, 93440401, 93440405, 93440419, 93440493, 378097201, 57664024188, 18860012101, 378035101, 378035110, 781139101, 781139113, 51079073301, 51079073320, 55289015797, 61392026315, 61392026345, 61392026356, 247127220, 247127230, 247127260, 247127290, 378025701, 378025710, 781139201, 781139213, 904592361, 43063055130, 51079073401, 51079073420, 52959051810, 54868009101, 58016071600, 58016071612, 58016071615, 58016071620, 58016071630, 58864022215, 61392026615, 61392026645, 61392026656, 63739012110, 63874083060, 378033401, 406208001, 781139701, 781139713, 904641361, 51079043101, 51079043120, 57866388001, 57866388002, 61392027530, 61392027531, 61392027532, 61392027539, 61392027545, 61392027551, 61392027554, 61392027556, 61392027560, 61392027590, 61392027591, 63874065610, 63874065614, 63874065615,

© 2024 Rodriguez MI et al. *JAMA Network Open.*

		63874065620, 63874065630, 63874065660, 68084024901, 68084024911, 68382008001, 68382008010, 247081703, 247081707, 247081730, 378021401, 378021410, 781139301, 781139313, 904592461, 51079073501, 51079073520, 57866387801, 58016002900, 58016002930, 58016002960, 58016002990, 58864073142, 61392026915, 61392026945, 61392026956, 61392026991, 63874049301, 63874049390, 93960412, 93960423, 121058104, 121058105, 603129054, 54838050115, 54838050140, 58016869101, 378033501, 406208101, 781139801, 57866388101, 61392012145, 61392012151, 61392012154, 61392012191, 63874100401, 68084025011, 68084025021, 68382008101, 247183903, 247183907, 247183908, 247183916, 378032701, 378032710, 781139601, 781139610, 781139613, 904592561, 21695065360, 35356009560, 51079073601, 51079073620, 51079073630, 51079073656, 54569288302, 57866387901, 60687016101, 60687016111, 61392027245, 61392027254, 61392027256, 61392027291, 63739012310, 63874064601, 63874064612, 63874064614, 63874064615, 63874064620, 63874064628, 63874064630, 63874064660, 68382007901, 68382007910, 78059520, 43068010102, 78060208, 43068011304, 78060020, 43068011002, 78060120, 43068011202, 78059620, 43068010202, 78059720, 43068010402, 78059820, 43068010602, 78059920, 43068010802, 182130601, 378701001, 527139501, 591037001, 10135058601, 51079090120, 52544049501, 68084000211, 68084000221, 182130701, 378702501, 527139601, 591037101, 10135058701, 51079090201, 51079090220, 52544049601, 68084000321, 182130501, 378700501, 527139401, 591036901, 10135058501, 52544036901, 52544036910, 182130801, 378705001, 527139701, 591037201, 10135058801, 51079090301, 51079090320, 52544037210, 52544049701, 68084000421, 63402031230, 63402030230, 63402030401, 63402030410, 63402030430, 63402030610, 63402030630, 63402306100, 63402030801, 63402030810, 63402030830, 64720022910, 64720023010, 64720022810, 2411701, 2411704, 2411730, 2411733, 93577001, 93577010, 93577056, 247184203, 247184207, 378545410, 378545491, 378545493, 904628561, 904637661, 13411013701, 13411013703, 13411013706, 13411013709, 13411013715, 13668016901, 13668016910, 13668016930, 21695014215, 33342007007, 33342007015, 42292001401, 42292001420, 43598016605, 43598016630, 49999060615, 49999060630, 51079015420, 52343004130, 52343004199, 54868394100, 55111016605, 55111016630, 55289021330, 58864096815, 59762010801, 60505311300, 60505311303, 60505311308, 62756055418, 62756055483, 62756055488, 65862056430, 65862056499, 66105015503, 66105015506, 66105015509,

© 2024 Rodriguez MI et al. *JAMA Network Open.*

		66105015515, 66105015545, 66993005005, 66993005030, 66993046305, 66993046330, 66993068330, 68084052701, 68084052711, 68084074001, 2445401, 2445485, 93524665, 378551193, 13668008830, 33342008407, 33342008411, 35356032030, 49884032155, 50268061613, 55111026379, 55111026381, 59746030732, 60505327600, 60505327603, 62756075464, 65862065703, 66993005430, 66993046730, 66993068738, 2441501, 2441504, 2441530, 2441533, 93577101, 93577110, 93577156, 378552210, 378552291, 378552293, 904628606, 13411013801, 13411013803, 13411013806, 13411013809, 13411013815, 13668017001, 13668017010, 13668017030, 33342007107, 33342007115, 35356031730, 42292001501, 42292001520, 51079015520, 52343004230, 52343004299, 55111016705, 55111016730, 59762010901, 60505311400, 60505311403, 60505311408, 62756055518, 62756055583, 62756055588, 65862056530, 65862056599, 66105056201, 66105056203, 66105056206, 66105056209, 66105056215, 66993005105, 66993005130, 66993046405, 66993046430, 66993068430, 68084052801, 68084052811, 2445501, 2445585, 93524765, 378551293, 13668008930, 33342008507, 33342008511, 35356032130, 49884032255, 50268061713, 55111026481, 59746030832, 60505327700, 60505327703, 60687020821, 62756075564, 65862065803, 66993005530, 66993046830, 66993068838, 2411201, 2411204, 2411230, 2411233, 93576701, 93576710, 93576756, 378515710, 378515791, 378515793, 904628361, 13411013401, 13411013403, 13411013406, 13411013409, 13411013415, 13668016601, 13668016610, 13668016630, 33342006707, 33342006744, 33358037260, 35356031630, 42292001201, 42292001220, 51079015201, 51079015220, 52343003830, 52343003899, 52959057930, 54868425500, 54868425501, 55111016305, 55111016330, 55887027230, 58864070430, 59762010501, 60505311000, 60505311003, 60505311008, 62756055118, 62756055183, 62756055188, 63629363301, 65862056130, 65862056199, 66105013301, 66105013303, 66105013306, 66105013315, 66105013345, 66993004705, 66993004730, 66993046005, 66993046030, 66993068030, 68084052501, 68084052511, 2442001, 2442004, 2442030, 2442033, 93510501, 93510505, 93510556, 378571310, 378571391, 378571393, 904628706, 13411013901, 13411013903, 13411013906, 13411013909, 13411013915, 13668017101, 13668017110, 13668017130, 21695014430, 33342007207, 33342007215, 35356031830, 42292001601, 42292001620, 51079015620, 52343004330, 52343004399, 55111016805, 55111016830, 55289049930, 59762011001, 60505314000, 60505314003, 60505314008, 62756055618, 62756055683, 62756055688, 65862056630,

		65862056699, 66105056301, 66105056303, 66105056306, 66105056309, 66105056315, 66993005205, 66993005230, 66993046505, 66993046530, 66993068530, 68084052901, 68084052911, 2445601, 2445685, 93524865, 378551393, 13668009030, 33342008607, 33342008611, 35356032230, 49884032355, 50268061813, 55111026581, 59746030932, 60505327800, 60505327803, 60687021921, 62756075764, 65862065903, 66993005630, 66993046930, 66993068938, 2411501, 2411530, 2411533, 93576801, 93576810, 93576856, 247186308, 247186314, 378521210, 378521291, 378521293, 904628461, 904637761, 13411013501, 13411013503, 13411013506, 13411013509, 13411013515, 13668016701, 13668016710, 13668016730, 21695014030, 33342006807, 33342006844, 42292001301, 42292001320, 43598016405, 43598016430, 49999037930, 49999073915, 49999073930, 51079015320, 52343003930, 52343003999, 54868425400, 54868425401, 55111016405, 55111016430, 58864076815, 59762010601, 60505311100, 60505311103, 60505311108, 62756055218, 62756055283, 62756055288, 65862056230, 65862056299, 66105013203, 66105013206, 66105013209, 66105013215, 66105013245, 66993004805, 66993004830, 66993046105, 66993046130, 66993068130, 68084052601, 68084052611, 68084072301, 2445301, 2445385, 93524565, 378551093, 13668008630, 33342008307, 33342008311, 35356031930, 49884032055, 50268061513, 55111026281, 59746030632, 60505327500, 60505327503, 60687018621, 62756075164, 65862065603, 66993005330, 66993046630, 66993068638, 2411601, 2411604, 2411630, 2411633, 93576901, 93576910, 93576956, 378533510, 378533591, 378533593, 13411013601, 13411013603, 13411013606, 13411013609, 13411013615, 13668016801, 13668016810, 13668016830, 33342006907, 33342006944, 43598016505, 43598016530, 52343004030, 52343004099, 54868533600, 55111016505, 55111016530, 58864089630, 59762010701, 60505311200, 60505311203, 60505311208, 62756055318, 62756055383, 62756055388, 65862056330, 65862056399, 66105055401, 66105055403, 66105055406, 66105055409, 66105055415, 66993004905, 66993004930, 66993046205, 66993046230, 66993068230, 378397893, 591369230, 10147095103, 50458055401, 378397993, 591369319, 591369330, 10147095201, 10147095203, 35356045030, 50458055001, 50458055010, 378398093, 591369419, 591369430, 10147095301, 10147095303, 21695045530, 35356050230, 50458055101, 50458055110, 378398193, 591369519, 591369530, 10147095401, 10147095403, 50458055201, 50458055210, 49884034701, 57844015101, 49884034801, 57844018701, 57844019801, 54022120, 54022125, 93816201, 93816210,

© 2024 Rodriguez MI et al. *JAMA Network Open.*

		310027110, 310027139, 781534401, 904627961, 13411012801, 13411012803, 13411012806, 13411012809, 13411012815, 13668015001, 16590052030, 16590052060, 16590052072, 16590052090, 16714037701, 16714037702, 16714045401, 16714045402, 16729014701, 16729014717, 18837014130, 21695011915, 23490708903, 42291069710, 42291069790, 47335090418, 47335090488, 49999060200, 49999060215, 49999060230, 50268063215, 54569569101, 54868425700, 54868425701, 54868425702, 54868425703, 54868425704, 55111018601, 55289018730, 58864095930, 60505313301, 60505313308, 63739066510, 65862049101, 65862049199, 66105014101, 66105014103, 66105014106, 66105014109, 66105014110, 67544019615, 67877025001, 67877025010, 67877025033, 67877025038, 68001018400, 68001018408, 68084053201, 68084053211, 68180044701, 68180044703, 310028139, 310028160, 54022220, 54022225, 93816301, 93816310, 310027210, 310027239, 781534601, 904628061, 13411012901, 13411012903, 13411012906, 13411012909, 13411012915, 13668015101, 16714037801, 16714045501, 16729014800, 16729014801, 16729014817, 21695012015, 23490709002, 42291069810, 42291069890, 47335090518, 47335090588, 49999095130, 50268063315, 54868427200, 54868427201, 55111018901, 55289044730, 58016004600, 58016004630, 58016004660, 58016004690, 58864096130, 60505313501, 60505313508, 63739067710, 65862049301, 65862049399, 66105014201, 66105014203, 66105014206, 66105014209, 66105014210, 67544028130, 67877024601, 67877024610, 67877024638, 68001018200, 68001018203, 68084053301, 68084053311, 68180044801, 68180044802, 310028239, 310028255, 310028260, 35356044030, 54022020, 54022025, 54022031, 93206301, 93206310, 93816101, 93816110, 247234100, 247234130, 247234160, 247234177, 247234190, 310027510, 310027534, 310027539, 378101101, 378101110, 781534301, 904627761, 12280003030, 13411012701, 13411012703, 13411012706, 13411012709, 13411012715, 13668014801, 13668014810, 16590052130, 16590052160, 16590052172, 16590052190, 16714037501, 16714037502, 16714045201, 16714045202, 16729014501, 16729014517, 18837014330, 18837014360, 21695011715, 33358032030, 33358032060, 42291069510, 47335090218, 47335090288, 49999060300, 49999060315, 50268063015, 54569570700, 54868496100, 54868496101, 54868496102, 54868496103, 54868496104, 55111024901, 55111024905, 55289087230, 58864073830, 58864073860, 60505313001, 60505313008, 65862048901, 65862048999, 66105014001, 66105014003, 66105014006, 66105014009, 66105014010, 67544015530, 67877024201, 67877024210,

© 2024 Rodriguez MI et al. *JAMA Network Open.*

		67877024233, 67877024238, 68001018500, 68001018508, 68084053001, 68084053011, 68180044501, 68180044503, 54022320, 54022321, 93816401, 93816410, 310027439, 310027460, 781534701, 904639861, 12280004160, 13411013001, 13411013003, 13411013006, 13411013009, 13411013015, 13668015201, 13668015260, 16714037901, 16714045601, 16729014900, 16729014901, 16729014912, 16729014917, 35356020460, 42291069910, 42291069960, 47335090618, 47335090686, 47335090688, 50268063415, 54868548400, 54868548401, 55111019060, 58864088830, 60505313706, 63739069010, 65862049460, 65862049499, 67877024710, 67877024738, 67877024760, 68001018300, 68001018303, 68001018306, 68084053401, 68084053411, 68180044901, 68180044902, 68180044907, 310028339, 310028355, 310028360, 35356044130, 54023020, 54023025, 93816501, 93816510, 310027910, 310027939, 781534801, 904628161, 13668015301, 16714038001, 16714045701, 16729015000, 16729015001, 16729015016, 35356025130, 42291070010, 42291070090, 47335090718, 47335090788, 50268063515, 55111060601, 60505313901, 65862049501, 65862049505, 67877024801, 67877024838, 68001018100, 68001018103, 68084053501, 68084053511, 68180045001, 68180045002, 310028439, 310028460, 35356044230, 54022920, 54022925, 54022931, 93816601, 93816610, 310027810, 310027834, 310027839, 781534201, 904627861, 13668014901, 13668014910, 16590051930, 16590051960, 16590051972, 16590051990, 16714037601, 16714037602, 16714045301, 16714045302, 16729014601, 16729014617, 21695011815, 35356008600, 35356008630, 42291069610, 47335090318, 47335090388, 50268063115, 54868558100, 54868558101, 54868558102, 55111016901, 55111016905, 60505313201, 60505313208, 65862049001, 65862049099, 67877024901, 67877024910, 67877024938, 68001018000, 68001018008, 68084053101, 68084053111, 68180044601, 68180044603, 310028039, 310028060, 93022105, 93022106, 247231924, 378350205, 378350291, 603568320, 603568328, 904597361, 904635761, 13107011905, 13107011960, 13668003501, 13668003505, 13668003560, 13668003574, 16252055860, 27241000206, 27241000250, 43547033906, 43547033950, 49999077560, 50458030101, 50458030104, 50458030106, 50458030150, 50458059010, 50458059050, 50458059060, 51079046001, 51079046020, 51991031606, 52343000305, 52343000360, 55111020105, 55111020160, 58016005000, 58016005030, 58016005060, 58016005090, 60505258400, 60505258405, 60505258406, 64679055302, 64679055304, 65862011905, 65862011960, 68084027001, 68084027011, 68382011205, 68382011214, 378604228, 49884021252,

© 2024 Rodriguez MI et al. *JAMA Network Open.*

		49884021255, 93022505, 93022506, 378350505, 378350591, 603568420, 603568428, 603568432, 904597461, 904635861, 13107012005, 13107012060, 13668003601, 13668003605, 13668003660, 13668003674, 16252055960, 27241000306, 27241000350, 33358031830, 33358031860, 43547034006, 43547034050, 49999091115, 50268069515, 50458030201, 50458030206, 50458030250, 50458059110, 50458059150, 50458059160, 51079046101, 51079046120, 51991031706, 52343000405, 52343000460, 52959021960, 55111020205, 55111020260, 55289046330, 58016004900, 58016004930, 58016004960, 58016004990, 60505258500, 60505258505, 60505258506, 64679055402, 64679055404, 65862012005, 65862012060, 66105012606, 68084027101, 68084027111, 68115082715, 68382011305, 68382011314, 378604328, 378604393, 781531008, 49884031152, 49884031155, 49884031191, 50458039528, 50458039530, 50458060128, 55111020781, 59746001032, 68382015406, 93724005, 93724006, 247184708, 247184710, 247184716, 247184718, 378351105, 378351191, 603568520, 603568528, 603568532, 904597561, 904635961, 13107012105, 13107012160, 13411012301, 13411012303, 13411012306, 13411012309, 13411012315, 13668003701, 13668003705, 13668003760, 13668003774, 16252056060, 21695008960, 21695011315, 27241000106, 27241000150, 43063025430, 43063025460, 43353040330, 43353043230, 43547034106, 43547034150, 49999063315, 50268069615, 50458030001, 50458030006, 50458030050, 50458059210, 50458059250, 50458059260, 51079046201, 51079046220, 51079046230, 51079046256, 51991031806, 52343000505, 52343000560, 54569414001, 55111020305, 55111020360, 55289049130, 58864003830, 60505258600, 60505258605, 60505258606, 63739054610, 64679055502, 64679055504, 65862012105, 65862012160, 66105047206, 67544021015, 68084027201, 68084027211, 68382011405, 68382011414, 378604428, 378604493, 781531108, 49884031552, 49884031555, 49884031591, 50458031528, 50458031530, 50458060228, 51079034501, 51079034505, 55111020881, 55111020891, 59746002022, 68382015506, 54006344, 93616931, 603942445, 23155031751, 42769133101, 50458030503, 50458059601, 51991071641, 55111057930, 60505038001, 64679069201, 65162067384, 65862016730, 93724105, 93724106, 247184003, 247184007, 378351205, 378351291, 603568620, 603568628, 603568632, 904597661, 904636061, 13107012205, 13107012260, 13411012401, 13411012403, 13411012406, 13411012409, 13411012415, 13668003801, 13668003805, 13668003860, 13668003874, 16252056160, 21695009560, 27241000406, 27241000450, 43547034206, 43547034250, 49999063430, 49999063460,

© 2024 Rodriguez MI et al. *JAMA Network Open.*

		50268069715, 50458032001, 50458032006, 50458032050, 50458059310, 50458059350, 50458059360, 51079046301, 51079046320, 51079046330, 51079046356, 51991031906, 52343000605, 52343000660, 52959091630, 52959091660, 52959094130, 55111020405, 55111020460, 55289046530, 60505258700, 60505258705, 60505258706, 63739054710, 64679055702, 64679055704, 65862012205, 65862012260, 66105012301, 66105012303, 66105012306, 66105012309, 66105012315, 68084027301, 68084027311, 68382011505, 68382011514, 378604528, 781531208, 49884040191, 50458032528, 50458060328, 51079034601, 51079034605, 55111020981, 59746003022, 68382015606, 93724205, 93724206, 247180503, 247180507, 247180530, 247180560, 378351305, 378351391, 603568920, 603568928, 904597761, 904636161, 13107012305, 13107012360, 13411012503, 13668003901, 13668003905, 13668003960, 13668003974, 16252056260, 21695011530, 21695011560, 27241000506, 27241000550, 35356010660, 43547034306, 43547034350, 50268069815, 50458033001, 50458033006, 50458033050, 50458059410, 50458059450, 50458059460, 51079046401, 51079046420, 51079046430, 51079046456, 51991032006, 52343000705, 52343000760, 55111020505, 55111020560, 60505258800, 60505258805, 60505258806, 64679057102, 64679057104, 65862012305, 65862012360, 66105012401, 66105012403, 66105012406, 66105012409, 66105012415, 68084027401, 68084027411, 68115078330, 68382011605, 68382011614, 378604628, 781531308, 49884040291, 50458033528, 50458060428, 51079034701, 51079034705, 55111047081, 59746004022, 93724306, 378351491, 603568820, 603568828, 904597861, 904636261, 13107012460, 13411012603, 13668004001, 13668004005, 13668004060, 13668004064, 16252056360, 27241000606, 27241000650, 43353040115, 43353041260, 43353045015, 43547034406, 43547034450, 50268069915, 50458035001, 50458035006, 50458059510, 50458059560, 51079046501, 51079046520, 51079046530, 51079046556, 51991032106, 52343000860, 55111020660, 55289051930, 60505258900, 60505258906, 64679057202, 64679057204, 65862012460, 66105012501, 66105012503, 66105012506, 66105012509, 66105012515, 68084027701, 68084027711, 68382011705, 68382011714, 378604728, 781531408, 49884040391, 50458035528, 50458060528, 55111047181, 59746005022, 49396041, 49396060, 378735005, 378735091, 781216460, 904626908, 21695006010, 21695006015, 21695006045, 49999062060, 51079035101, 51079035116, 55111025660, 58864096030, 59762200101, 60505252806, 63874100701, 63874100706, 64679099102, 64679099104, 68001013606, 68084010309,

© 2024 Rodriguez MI et al. *JAMA Network Open.*

		68084058109, 68084058111, 68180033107, 49397041, 49397060, 378735105, 378735191, 781216660, 904627008, 21695006115, 21695006145, 35356027460, 51079035201, 51079035216, 55111025760, 58864096230, 59762200201, 60505252906, 64679099202, 64679099204, 68001013706, 68084010409, 68084058209, 68084058211, 68180033207, 49398041, 49398060, 378735205, 378735291, 781216760, 904627108, 21695006260, 49999062160, 51079035301, 51079035316, 55111025860, 58864095830, 59762200301, 60505253006, 63874100606, 64679099302, 64679099304, 68001013806, 68084010509, 68084058309, 68084058311, 68180033307, 49399041, 49399060, 378735305, 378735391, 781216860, 904627208, 21695006360, 35356009760, 51079035401, 51079035416, 55111025960, 58864096430, 59762200401, 59762200402, 60505253106, 63874100806, 64679099402, 64679099404, 68001013906, 68084010609, 68084058409, 68084058411, 68180033407, 52011906, 52011990, 52214203, 52214204, 52214206, 456241011, 456241060, 456241063, 456240260, 52011806, 52011890, 52213903, 52213904, 456240560, 456240563, 182085963, 641139731, 641139735, 641139831, 641139835, 49727076010, 63323028110, 641049121, 641049125, 10019004301, 10019004344, 23155029442, 23155049731, 23155049742, 55390007701, 55390007710, 781171501, 781591301, 832030000, 832030010, 51079051801, 51079051820, 61392003932, 61392003945, 61392003951, 61392003954, 61392003956, 61392003960, 61392003990, 61392003991, 68084042001, 68084042011, 182047601, 182047610, 247184604, 247184608, 781171801, 781591601, 832030300, 832030301, 832030310, 832030389, 35356009890, 51079051601, 51079051620, 62584033101, 62584033111, 182047701, 182047710, 781171901, 781591701, 832030400, 832030401, 832030410, 832030489, 51079051701, 51079051720, 68084042201, 68084042211, 182047401, 182047410, 781171601, 781591401, 832030100, 832030101, 832030110, 832030189, 51079051901, 51079051920, 54868246400, 61392004045, 61392004054, 61392004056, 61392004091, 62584033001, 62584033011, 182047501, 182047510, 781171701, 781591501, 832030200, 832030201, 832030210, 832030289, 51079013001, 51079013020, 68084042101, 68084042111, 378600401, 378600405, 527178801, 527178805, 781143601, 781143613, 17236048911, 49884006101, 49884006105, 51079048501, 51079048520, 57866440601, 57866440602, 57866440603, 57866440604, 61392005745, 61392005751, 61392005754, 61392005791, 378609701, 378609705, 527179101, 527179105, 781143901, 17236049211, 49884006401, 49884006405, 49884006410, 51079048801, 51079048820, 57866440801,

© 2024 Rodriguez MI et al. *JAMA Network Open.*

		57866440802, 57866440803, 57866440804, 61392006030, 61392006031, 61392006032, 61392006039, 61392006045, 61392006051, 61392006054, 61392006060, 61392006090, 61392006091, 68084095025, 68084095095, 378600901, 378600905, 527178901, 527178905, 781143701, 17236049011, 49884006201, 49884006205, 51079048601, 51079048620, 57866440701, 57866440702, 57866440703, 57866440704, 58864080240, 61392005831, 61392005832, 61392005839, 61392005845, 61392005851, 61392005854, 61392005860, 61392005890, 61392005891, 93968616, 121065402, 121065416, 378607401, 378607405, 527179001, 781143801, 17236049111, 49884007601, 49884007605, 51079048701, 51079048720, 57866440501, 57866440502, 57866440503, 57866440504, 61392005930, 61392005931, 61392005932, 61392005939, 61392005945, 61392005951, 61392005954, 61392005960, 61392005990, 61392005991, 67544093112, 68084084621, 68084084625, 68084084695, 85029605, 121065304, 85007705, 603506321, 603509321, 781104901, 54738055301, 54738055302, 68084084221, 68084084225, 68084084295, 603506021, 603506028, 603509021, 603509028, 781104601, 781104610, 781104613, 54738055001, 61392008245, 61392008254, 61392008256, 61392008291, 68084083021, 68084083025, 68084083095, 85094005, 490009100, 490009130, 490009160, 490009190, 603506121, 603506128, 603509121, 603509128, 781104701, 781104710, 781104713, 21695041460, 54738055101, 54738055102, 57866413201, 57866413202, 57866413203, 61392008145, 61392008154, 61392008156, 61392008191, 68084060201, 68084060211, 85031305, 603506221, 603506228, 603509221, 603509228, 781104801, 781104810, 781104813, 35356009660, 52959094060, 54738055201, 54738055202, 57866413301, 57866413302, 57866413303, 61392014245, 61392014251, 61392014254, 61392014291, 68084060701, 68084060711, 93965201, 247049730, 378511001, 555052204, 781502101, 10135036801, 16590032710, 21695057230, 23490650903, 33358030020, 33358030030, 33358030060, 43063016004, 43063016006, 51079054201, 51079054220, 51655029489, 52959047602, 52959047610, 52959047615, 52959047620, 52959047624, 52959047630, 52959047660, 54569035500, 54569035501, 54569035502, 54569035503, 54868108200, 54868108201, 54868108202, 54868108203, 54868108204, 54868108205, 54868108206, 55045112602, 55045112604, 55045112606, 55045112607, 55045112608, 55289022404, 55289022410, 55289022412, 55289022415, 55289022420, 55289022497, 55887061904, 55887061920, 55887061960, 57866629801, 58016070600, 58016070602, 58016070603, 58016070608,

© 2024 Rodriguez MI et al. *JAMA Network Open.*

		58016070630, 58016070660, 58016070690, 58864064442, 59746011506, 59746011510, 63629133501, 63629133502, 63629133503, 63874049001, 63874049006, 63874049008, 63874049010, 63874049012, 63874049015, 63874049020, 63874049028, 63874049030, 63874049060, 66213011710, 66336092115, 66993081002, 67544039206, 68084083921, 93964301, 247082505, 247082520, 378510501, 440219012, 440819012, 781502001, 10135036701, 23490651201, 23490651202, 33358029920, 33358029930, 43063035306, 51079054101, 51079054119, 51079054120, 51655009387, 52959051110, 52959051115, 54569035001, 54569035002, 54569035003, 54569035005, 54868472100, 54868472101, 54868472102, 54868472103, 55045169602, 55289056810, 55289056812, 55289056820, 55289056830, 57866629901, 57866629902, 58016032612, 58016032660, 58016032690, 58864070201, 59746011306, 59746011310, 60429016202, 63629184101, 63874052530, 66213011510, 66336043406, 66336043410, 66993080502, 247080007, 247080014, 247080030, 378061201, 378061210, 51079056501, 51079056520, 53489014801, 53489014810, 57866104201, 61392046215, 61392046245, 61392046256, 61392046291, 378061801, 51079058001, 51079058020, 53489050001, 61392046530, 61392046531, 61392046532, 61392046539, 61392046545, 61392046551, 61392046554, 61392046560, 61392046590, 247080107, 247080114, 247080130, 378061401, 378061410, 51079056601, 51079056620, 53489014901, 53489014910, 54868006700, 61392046345, 61392046354, 61392046391, 247080207, 247080214, 247080230, 378061601, 378061610, 51079056701, 51079056720, 53489015001, 53489015010, 61392046430, 61392046431, 61392046432, 61392046439, 61392046445, 61392046451, 61392046454, 61392046460, 61392046490, 61392046491, 378240101, 781103001, 832049411, 51079057201, 51079057220, 378241001, 378241005, 781103601, 832049711, 51079057501, 51079057520, 61392015132, 61392015145, 61392015151, 61392015154, 61392015191, 378240201, 781103201, 832049511, 51079057301, 51079057320, 61392015232, 61392015245, 61392015251, 61392015254, 61392015260, 61392015291, 247184908, 247184916, 378240501, 378240505, 781103401, 832049611, 51079057401, 51079057420, 61392015430, 61392015431, 61392015432, 61392015439, 61392015445, 61392015451, 61392015454, 61392015460, 61392015490, 61392015491, 574722612, 713013510, 713013512, 23490617401, 52959035506, 52959035512, 54569472000, 54569472002, 55045240002, 55289011902, 55289011906, 55887022004, 55887022012, 66213020012, 378100101, 781222601, 17236046511, 51079058601, 51079058620,

© 2024 Rodriguez MI et al. *JAMA Network Open.*

		61392078830, 61392078831, 61392078832, 61392078839, 61392078845, 61392078851, 61392078854, 61392078860, 61392078890, 61392078891, 378501001, 378501010, 781222901, 17236046811, 51079058901, 51079058920, 61392079130, 61392079131, 61392079132, 61392079139, 61392079145, 61392079151, 61392079154, 61392079160, 61392079190, 61392079191, 378200201, 378200210, 781222701, 17236046611, 51079058701, 51079058720, 61392078930, 61392078931, 61392078932, 61392078939, 61392078945, 61392078951, 61392078954, 61392078960, 61392078990, 61392078991, 378300501, 378300510, 781222801, 17236046711, 21695016130, 51079058801, 51079058820, 61392079030, 61392079031, 61392079032, 61392079039, 61392079045, 61392079051, 61392079054, 61392079060, 61392079090, 61392079091, 500901470, 500902754, 500902799, 500903490, 500902993, 500903917, 500903918, 500902959, 500906052, 332610852, 623320148, 467080148, 467080303, 467080304, 467080305, 680840567, 680840655, 680840640, 680840942, 680840953, 625590340, 675440534, 675440946, 716100557, 605052504, 691890020, 691890023, 691890526, 636294170, 713350120, 636297730, 713351006, 636298282, 713351543, 636291916, 636291917, 636291955, 636299190, 317220544, 317220545, 317220546, 551544920, 551544941, 551544960, 551547456, 681510245, 681510639, 581180545, 670460430, 670460431, 670460432, 670460397, 684620220, 684620221, 684620222, 684620223, 604290267, 604290423, 604290424, 231550293, 231550763, 659775044, 659775045, 659775046, 591150135, 540020, 540021, 542526, 542527, 542531, 544527, 548526, 548527, 548528, 548531, 9046204, 9046205, 9046136, 637390265, 510790180, 510790142, 3781300, 3781450, 6151311, 6152304, 6155593, 6158302, 680712186, 680712714, 430630196, 430630901, 727890171, 727890172, 727890173, 727890174, 548681335, 548683632, 548685239, 548685340, 687887164, 631870304, 712050238, 493490156, 493490691, 242360192, 242360736, 521250181, 521250208, 521250282, 521250304, 617860440, 617860559, 617860620, 242360081, 705180150, 705180360, 242360082, 705180377, 705180437, 705180608, 705180629, 705180668, 705180904, 705181434, 705181481, 705181567, 705181753, 705182086, 705182312, 705182345, 705182699, 649800205, 649800278, 165900781, 538080643, 538080704, 538080705, 538080884, 627560430, 647250545, 647250544, 647252527, 1431300, 1431277, 1433188, 1433189, 1433190, 551544952, 543527, 609861019, 680940757, 548529, 500903027, 503830792, 606870262, 675440479, 433530085, 433530279, 178560675, 178560152,

© 2024 Rodriguez MI et al. *JAMA Network Open.*

		178560792, 691890852, 693870107, 694520150, 636292454, 576640124, 551545361, 551549430, 551545683, 551541575, 621350196, 670460807, 670460808, 721890010, 721890267, 719300057, 604290246, 5275407, 5275250, 637390253, 637390086, 637390251, 604320621, 510790298, 6151325, 6158205, 680712702, 6031841, 1210675, 1214675, 1211350, 501110852, 680940193, 680940701, 216950417, 493490304, 617860527, 705180079, 705180730, 705181968, 627560873, 939633, 8321008, 8320310, 5910426, 500901391, 500901425, 500901473, 500902129, 500902130, 500902131, 500902466, 500902474, 500902814, 500902825, 500903098, 500903103, 500903129, 500903171, 500903278, 500902992, 500903914, 500904939, 500905567, 500905827, 500905828, 500905850, 162520597, 162520598, 2281410, 2281422, 2281435, 2281453, 2281580, 2281638, 728880025, 728880026, 728880027, 728880028, 332610647, 332610648, 332610946, 272410183, 272410184, 272410185, 272410186, 623320095, 623320096, 623320037, 623320038, 623320039, 623320040, 467080095, 467080096, 467080274, 467080275, 467080276, 467080277, 680840318, 680840319, 680840320, 680840321, 680840335, 680840810, 651620949, 651620956, 651620958, 651620986, 651620988, 651620990, 1151526, 1151527, 1151528, 1151529, 1159939, 1159940, 1159941, 1159942, 699180350, 699180360, 699180370, 699180380, 605052663, 605052664, 605052665, 605052680, 678770164, 678770165, 678770166, 678770167, 764200182, 658620227, 658620228, 658620229, 658620230, 658620361, 658620362, 691890188, 422910366, 422910367, 422910368, 422910369, 422910443, 422910444, 422910445, 422910446, 422910447, 422910448, 422910353, 422910354, 502680461, 502680462, 502680463, 502680464, 636294169, 636294171, 636294183, 636294697, 713350010, 713350044, 713350057, 713350276, 713350706, 713350830, 713351110, 713351179, 636298283, 713351469, 713351579, 713351748, 713351919, 713352071, 658410682, 658410683, 658410684, 658410685, 658410686, 658410687, 658410689, 658410690, 707711514, 707711515, 707711516, 707711517, 707711518, 707711519, 551548274, 551548275, 551545387, 551545396, 551548129, 551548137, 551548142, 551542330, 551542331, 551542065, 681514249, 690970148, 690970149, 690970151, 690970152, 581184132, 581184131, 581182148, 581181111, 162520596, 709340975, 619190802, 619190777, 721890063, 619190534, 435980550, 435980551, 435980552, 435980553, 551110220, 551110221, 551110222, 551110223, 551110225, 551110226, 551110428, 551110717, 551110718, 551110719, 551110720,

© 2024 Rodriguez MI et al. *JAMA Network Open.*

		551110419, 684620228, 684620229, 684620243, 684620244, 684620245, 684620246, 514070060, 514070061, 514070062, 514070063, 597622040, 597622041, 597622042, 597622043, 597622460, 597622461, 765191150, 765191151, 765191165, 591150065, 591150066, 591150067, 591150068, 1439964, 1439965, 1439966, 1439967, 597460245, 597460246, 597460247, 597460248, 597460311, 597460312, 353560448, 353560619, 557000626, 557000683, 681800250, 681800251, 681800252, 681800253, 9047007, 9047008, 762370262, 762370263, 650840179, 637390448, 637390516, 637390517, 637390670, 637390179, 510790498, 510790499, 510790865, 510790866, 3784251, 3784252, 3784253, 3784254, 3786905, 3786925, 6157544, 6157545, 6157546, 6157547, 6157964, 6157975, 6158150, 6158267, 167140700, 167140701, 167140702, 167140703, 167140623, 167140624, 167140625, 167140626, 167140627, 167140893, 167140194, 167140195, 167140196, 167140197, 167140371, 167140372, 167140373, 167140374, 680712054, 680712729, 691020639, 498840484, 498840485, 498840486, 498840487, 498840880, 498840881, 498840882, 430630202, 430630203, 430630238, 430630259, 727890206, 548685921, 548685955, 548686078, 548686325, 687886868, 687887168, 687887178, 687887223, 687887278, 687887871, 687886822, 687889526, 687887544, 687887465, 687887647, 631870487, 631870744, 712050245, 216950107, 216950227, 216950228, 216950229, 242360943, 493490055, 493490349, 493490710, 493490727, 493490801, 493490971, 493490998, 521250087, 521250117, 521250154, 242360945, 493490857, 493490866, 493490900, 493490911, 521250280, 521250864, 521250939, 617860010, 617860220, 617860523, 617860614, 617860624, 617860625, 617860932, 617860999, 705180001, 705181153, 705181181, 705181479, 705181480, 705181523, 705181602, 705181908, 705181978, 705182019, 705182093, 705182108, 705182343, 705182406, 705182473, 705182503, 705182535, 705182563, 705182577, 705182697, 705182716, 705182755, 705182889, 705183043, 705183109, 705183139, 705183150, 705183187, 705183425, 165710785, 165710786, 649800400, 649800401, 530021512, 530021513, 530022512, 530022513, 7814040, 7815122, 7815123, 7815124, 7815125, 502280319, 502280320, 502280321, 502280322, 607600549, 607600050, 607600474, 607600543, 165900777, 165900802, 538080993, 538081003, 538081011, 516724130, 516724131, 516724132, 516724133, 516724139, 516724140, 930039, 930132, 930463, 930688, 937247, 937248, 136680045, 136680047, 136680048, 136680049, 136680266, 136680339, 136680340,

© 2024 Rodriguez MI et al. *JAMA Network Open.*

		136680341, 136680342, 647250111, 647250112, 293000111, 293000112, 293000113, 293000114, 504363995, 504363996, 504360149, 2450219, 2450220, 2450221, 2450222, 2450223, 2450224, 8320600, 8320601, 8320602, 8320603, 525360250, 525360251, 525360252, 525360253, 556480271, 556480272, 556480273, 556480274, 556480275, 646790271, 646790272, 646790273, 646790274, 646790275, 691170044, 691170045, 691170046, 691170047, 691170048, 691170049, 683820006, 683820007, 683820008, 683820009, 683820010, 683820011, 683820108, 683820109, 683820979, 683820980, 683820981, 683820982, 683820983, 683820984

© 2024 Rodriguez MI et al. *JAMA Network Open.*

eTable 4. *ICD-9* and *ICD-10* Codes for Mood Disorder Categories

Category	ICD9	ICD10
Anxiety and fear-related disorders	300.3, 300.5, 300.9, 308.0, 308.1, 308.2, 308.3, 308.4, 308.9, 313.0, 313.1, 313.3, 293.84, 300.00, 300.01, 300.02, 300.09, 300.10, 300.20, 300.21, 300.22, 300.23, 300.29, 300.89, 309.81, 313.21, 313.22, 313.23, 313.82, 313.83	F06.4, F40.00, F40.01, F40.02, F40.10, F40.11, F40.210, F40.218, F40.220, F40.228, F40.230, F40.231, F40.232, F40.233, F40.240, F40.241, F40.242, F40.243, F40.248, F40.290, F40.291, F40.298, F40.8, F40.9, F41.0, F41.1, F41.3, F41.8, F41.9, F93.0, F94.0
Bipolar and related disorders	296.7, 296.00, 296.01, 296.02, 296.03, 296.04, 296.05, 296.06, 296.10, 296.11, 296.12, 296.13, 296.14, 296.15, 296.16, 296.40, 296.41, 296.42, 296.43, 296.44, 296.45, 296.46, 296.50, 296.51, 296.52, 296.53, 296.54, 296.55, 296.56, 296.60, 296.61, 296.62, 296.63, 296.64, 296.65, 296.66, 296.80, 296.81, 296.82, 296.89, 296.90, 296.99	F06.30, F06.33, F06.34, F30.10, F30.11, F30.12, F30.13, F30.2, F30.3, F30.8, F30.9, F31.0, F31.10, F31.11, F31.12, F31.13, F31.2, F31.30, F31.31, F31.32, F31.4, F31.5, F31.60, F31.61, F31.62, F31.63, F31.64, F31.71, F31.73, F31.75, F31.77, F31.81, F31.89, F31.9, F34.0, F34.8, F34.81, F34.89, F34.9, F39
Depressive disorders	311, 300.4, 293.83, 296.20, 296.21, 296.22, 296.23, 296.24, 296.25, 296.26, 296.30, 296.31, 296.32, 296.33, 296.34, 296.35, 296.36, 301.10, 301.11, 301.12, 301.13	F06.31, F06.32, F32.0, F32.1, F32.2, F32.3, F32.4, F32.8, F32.81, F32.89, F32.9, F32.A, F33.0, F33.1, F33.2, F33.3, F33.41, F33.8, F33.9, F34.1

© 2024 Rodriguez MI et al. *JAMA Network Open.*

Schizophrenia spectrum and other psychotic disorders	316, 297.0, 297.1, 297.2, 297.3, 297.8, 297.9, 298.0, 298.1, 298.2, 298.3, 298.4, 298.8, 298.9, 293.81, 293.82, 295.00, 295.01, 295.02, 295.03, 295.04, 295.05, 295.10, 295.11, 295.12, 295.13, 295.14, 295.15, 295.20, 295.21, 295.22, 295.23, 295.24, 295.25, 295.30, 295.31, 295.32, 295.33, 295.34, 295.35, 295.40, 295.41, 295.42, 295.43, 295.44, 295.45, 295.50, 295.51, 295.52, 295.53, 295.54, 295.55, 295.60, 295.61, 295.62, 295.63, 295.64, 295.65, 295.70, 295.71, 295.72, 295.73, 295.74, 295.75, 295.80, 295.81, 295.82, 295.83, 295.84, 295.85, 295.90, 295.91, 295.92, 295.93, 295.94, 295.95, 301.20, 301.21, 301.22	F06.0, F06.1, F06.2, F20.0, F20.1, F20.2, F20.3, F20.5, F20.81, F20.89, F20.9, F21, F22, F23, F24, F25.0, F25.1, F25.8, F25.9, F28, F29

© 2024 Rodriguez MI et al. *JAMA Network Open.*

Suicidal ideation/attempt/intentional self-harm	E95.00, E95.01, E95.02, E95.03, E95.04, E95.05, E95.06, E95.07, E95.08, E95.09, E95.10, E95.11, E95.18, E95.20, E95.21, E95.28, E95.29, E95.30, E95.31, E95.38, E95.39, E95.4, E95.50, E95.51, E95.52, E95.53, E95.54, E95.55, E95.56, E95.57, E95.59, E95.6, E95.70, E95.71, E95.72, E95.79, E95.80, E95.81, E95.82, E95.83, E95.84, E95.85, E95.86, E95.87, E95.88, E95.89, E95.9	R45.851, R45.88, T14.91, T14.91XA, T36.0X2A, T36.1X2A, T36.2X2A, T36.3X2A, T36.4X2A, T36.5X2A, T36.6X2A, T36.7X2A, T36.8X2A, T36.92XA, T37.0X2A, T37.1X2A, T37.2X2A, T37.3X2A, T37.4X2A, T37.5X2A, T37.8X2A, T37.92XA, T38.0X2A, T38.1X2A, T38.2X2A, T38.3X2A, T38.4X2A, T38.5X2A, T38.6X2A, T38.7X2A, T38.802A, T38.812A, T38.892A, T38.902A, T38.992A, T39.012A, T39.092A, T39.1X2A, T39.2X2A, T39.312A, T39.392A, T39.4X2A, T39.8X2A, T39.92XA, T40.0X2A, T40.1X2A, T40.2X2A, T40.3X2A, T40.412A, T40.422A, T40.492A, T40.4X2A, T40.5X2A, T40.602A, T40.692A, T40.712A, T40.722A, T40.7X2A, T40.8X2A, T40.902A, T40.992A, T41.0X2A, T41.1X2A, T41.202A, T41.292A, T41.3X2A, T41.42XA, T41.5X2A, T42.0X2A, T42.1X2A, T42.2X2A, T42.3X2A, T42.4X2A, T42.5X2A, T42.6X2A, T42.72XA, T42.8X2A, T43.012A, T43.022A, T43.1X2A, T43.202A, T43.212A, T43.222A, T43.292A, T43.3X2A, T43.4X2A, T43.502A, T43.592A, T43.602A, T43.612A, T43.622A, T43.632A, T43.642A, T43.692A, T43.8X2A, T43.92XA, T44.0X2A, T44.1X2A, T44.2X2A, T44.3X2A, T44.4X2A, T44.5X2A, T44.6X2A, T44.7X2A, T44.8X2A, T44.902A, T44.992A, T45.0X2A, T45.1X2A, T45.2X2A, T45.3X2A, T45.4X2A, T45.512A, T45.522A, T45.602A, T45.612A, T45.622A, T45.692A, T45.7X2A, T45.8X2A, T45.92XA, T46.0X2A, T46.1X2A, T46.2X2A, T46.3X2A, T46.4X2A, T46.5X2A, T46.6X2A, T46.7X2A, T46.8X2A,

© 2024 Rodriguez MI et al. *JAMA Network Open.*

		T46.902A, T46.992A, T47.0X2A, T47.1X2A, T47.2X2A, T47.3X2A, T47.4X2A, T47.5X2A, T47.6X2A,T47.7X2A , T47.8X2A, T47.92XA, T48.0X2A, T48.1X2A, T48.202A, T48.292A, T48.3X2A, T48.4X2A, T48.5X2A, T48.6X2A, T48.902A, T48.992A, T49.0X2A, T49.1X2A, T49.2X2A, T49.3X2A, T49.4X2A, T49.5X2A, T49.6X2A, T49.7X2A, T49.8X2A, T49.92XA, T50.0X2A, T50.1X2A, T50.2X2A, T50.3X2A, T50.4X2A, T50.5X2A, T50.6X2A, T50.7X2A, T50.8X2A, T50.902A, T50.912A, T50.992A, T50.A12A, T50.A22A, T50.A92A, T50.B12A, T50.B92A, T50.Z12A, T50.Z92A, T51.0X2A, T51.1X2A, T51.2X2A, T51.3X2A, T51.8X2A, T51.92XA, T52.0X2A, T52.1X2A, T52.2X2A, T52.3X2A, T52.4X2A, T52.8X2A, T52.92XA, T53.0X2A, T53.1X2A, T53.2X2A, T53.3X2A, T53.4X2A, T53.5X2A, T53.6X2A, T53.7X2A, T53.92XA, T54.0X2A, T54.1X2A, T54.2X2A, T54.3X2A, T54.92XA, T55.0X2A, T55.1X2A, T56.0X2A, T56.1X2A, T56.2X2A, T56.3X2A, T56.4X2A, T56.5X2A, T56.6X2A, T56.7X2A, T56.812A, T56.892A, T56.92XA, T57.0X2A, T57.1X2A, T57.2X2A, T57.3X2A, T57.8X2A, T57.92XA, T58.02XA, T58.12XA, T58.2X2A, T58.8X2A, T58.92XA, T59.0X2A, T59.1X2A, T59.2X2A, T59.3X2A, T59.4X2A, T59.5X2A, T59.6X2A, T59.7X2A, T59.812A, T59.892A, T59.92XA, T60.0X2A, T60.1X2A, T60.2X2A, T60.3X2A, T60.4X2A, T60.8X2A, T60.92XA, T61.02XA, T61.12XA, T61.772A, T61.782A, T61.8X2A, T61.92XA, T62.0X2A, T62.1X2A, T62.2X2A, T62.8X2A, T62.92XA,

© 2024 Rodriguez MI et al. *JAMA Network Open.*

		T63.002A, T63.012A, T63.022A, T63.032A, T63.042A, T63.062A, T63.072A, T63.082A, T63.092A, T63.112A, T63.122A, T63.192A, T63.2X2A, T63.302A, T63.312A, T63.322A, T63.332A, T63.392A, T63.412A, T63.422A, T63.432A, T63.442A, T63.452A, T63.462A, T63.482A, T63.512A, T63.592A, T63.612A, T63.622A, T63.632A, T63.692A, T63.712A, T63.792A, T63.812A, T63.822A, T63.832A, T63.892A, T63.92XA, T64.02XA, T64.82XA, T65.0X2A, T65.1X2A, T65.212A, T65.222A, T65.292A, T65.3X2A, T65.4X2A, T65.5X2A, T65.6X2A, T65.812A, T65.822A, T65.832A, T65.892A, T65.92XA, T71.112A, T71.122A, T71.132A, T71.152A, T71.162A, T71.192A, T71.222A, T71.232A

© 2024 Rodriguez MI et al. *JAMA Network Open.*

Trauma- and stressor-related disorders	309.0, 309.1, 309.3, 309.4, 309.9, 309.22, 309.23, 309.24, 309.28, 309.29, 309.82, 309.83, 309.89	F43.0, F43.10, F43.11, F43.12, F43.20, F43.21, F43.22, F43.23, F43.24, F43.25, F43.29, F43.8, F43.9, F44.0, F44.1, F44.2, F44.4, F44.5, F44.6, F44.7, F44.81, F44.89, F44.9, F48.1, F94.1, F94.2
Other - Neurocognitive Disorders	797, 290.0, 290.3, 290.8, 290.9, 307.0, 307.3, 310.0, 310.2, 331.0, 331.1, 331.2, 290.10, 290.11, 290.12, 290.13, 290.20, 290.21, 290.40, 290.41, 290.42, 290.43, 294.10, 294.11, 294.20, 294.21, 301.50, 331.11, 331.19, 331.82	
Other - Neurodevelopmental Disorders	317, 319, 314.1, 314.2, 314.8, 314.9, 315.1, 315.2, 315.4, 315.5, 315.8, 315.9, 318.0, 318.1, 318.2, 299.00, 299.01, 299.10, 299.11, 299.80, 299.81, 299.90, 299.91, 314.00, 315.00, 315.01, 315.02, 315.09, 315.31, 315.32, 315.34, 315.35, 315.39	F93.8, F93.9, F98.21, F98.29, F98.3, F98.9

© 2024 Rodriguez MI et al. *JAMA Network Open.*

Other - Other Disorders	294.0, 294.1, 294.8, 294.9, 300.6, 301.3, 301.4, 301.6, 301.7, 306.0, 306.1, 306.2, 306.3, 306.4, 306.6, 306.7, 306.8, 306.9, 307.1, 307.9, 312.4, 312.8, 312.9, 293.89, 300.11, 300.12, 300.13, 300.14, 300.15, 300.16, 300.19, 300.81, 301.51, 301.59, 301.81, 301.82, 301.83, 301.84, 301.89, 306.50, 306.51, 306.52, 306.53, 306.59, 307.20, 307.21, 307.22, 307.23, 307.40, 307.41, 307.42, 307.43, 307.44, 307.45, 307.46, 307.47, 307.48, 307.49, 307.50, 307.51, 307.52, 307.53, 307.54, 307.59, 307.80, 307.81, 307.89, 309.21, 312.00, 312.01, 312.02, 312.03, 312.10,312.11, 312.12, 312.13, 312.20, 312.21, 312.22, 312.23, 312.30, 312.31, 312.32, 312.33, 312.34, 312.35, 312.39, 312.81, 312.82, 312.89, 313.81, 313.89, 314.01, 648.40, 648.41, 648.42, 648.43, 648.44, 293.0, 293.1, 293.9, 301.0, 301.9, 310.1, 310.8, 310.9, 313.9, 300.82, 310.81, 310.89	F55.0, F55.1, F55.2, F55.3, F55.4, F55.8, F91.0, F91.1, F91.2, F91.3, F91.8, F91.9, F50.00, F50.01, F50.02, F50.2, F50.8, F50.81, F50.82, F50.89, F50.9, F63.0, F63.1, F63.2, F63.3, F63.81, F63.89, F63.9, F53, F53.0, F53.1, F99, F42, F42.2, F42.3, F42.4, F42.8, F42.9, F68.10, F68.11, F68.12, F68.13, F68.8, F68.A, F06.8, F48.8, F48.9, F66, F65.0, F65.1, F65.2, F65.3, F65.4, F65.50, F65.51, F65.52, F65.81, F65.89, F65.9, F07.0, F07.9, F52.0, F52.1, F52.21, F52.22, F52.31, F52.32, F52.4, F52.5, F52.6, F52.8, R37, F45.0, F45.1, F45.20, F45.21, F45.22, F45.29, F45.41, F45.42, F45.8, F45.9, F60.0, F60.1, F60.2, F60.3, F60.4, F60.5, F60.6, F60.7, F60.81, F60.89, F60.9, F59, F69, F09

© 2024 Rodriguez MI et al. *JAMA Network Open.*

eTable 5. Sensitivity Analysis of Logistic Regression Model Estimates Compared With Linear Regression

	**Linear Regression DiD estimates** (Percentage points, 95% CI)	**Logistic Regression estimates** (OR, 95% CI)
**Mental Health Diagnosis**	4.1 (1.7, 6.5)	2.1 (2.1, 2.1)
**Treatment**	26.5 (15.5., 37.5)	12.5 (10.8, 14.5)

© 2024 Rodriguez MI et al. *JAMA Network Open.*

eTable 6. Perinatal Mental Health Diagnoses Among the Emergency Medicaid Population in Oregon and South Carolina Following Expansion of Prenatal Coverage and Postpartum Coverage

	Prenatal coverage expansion (n=22,039)				Postpartum coverage expansion (n=21,850)			
	Pre (n=13,311)		Post (n=8,728)		Pre (n=10,752)		Post (n=11,098)	
	N	%	N	%	N	%	N	%
**Mental Health Conditions[a]**	260	2.0	376	4.3	515	4.8	737	6.6
Anxiety	45	0.3	108	1.2	197	1.8	258	2.3
Bipolar disorder	6	0.0	5	0.1	11	0.1	20	0.2
Depression	147	1.1	148	1.7	242	2.3	294	2.6
Suicidal ideation/attempt/intentional self-harm	1	0.0	5	0.1	12	0.1	15	0.1
Trauma- and stressor-related disorders	7	0.1	38	0.4	145	1.3	216	1.9
Other[c]	217	1.6	251	2.9	52	0.5	148	1.3
**Postpartum Visit Attendance within 60 days**	131	1.0	629	7.2	1279	11.9	4258	38.4
**Received Any Talk Therapy[b]**	0	0.0	59	15.7	85	16.5	161	21.2
**Received Any Pharmacotherapy[b]**	1	0.4	15	4.0	63	12.2	55	7.5

[a] Categories of mental health conditions are not mutually exclusive (i.e., they will add up to more than any mood disorder %)
[b] Denominator for any talk therapy or any pharmacotherapy was those diagnosed with any mood disorder prenatally or within 60 days postpartum.
[C] Mental health conditions captured in the Other category are listed in Supplemental Table 3

© 2024 Rodriguez MI et al. *JAMA Network Open.*

eTable 7. Changes in Mood Disorder Diagnosis and Treatment Among the Emergency Medicaid Population Following Expansion of Postpartum Care Among Latina Individuals Only, 2016-2020

	**Women, No. (%)**						
	**Treatment (Oregon)**			**Comparison (South Carolina)**			
**Primary Outcomes**	**Pre-policy (n = 4,815)**	**Post-policy (n = 4,765)**	**Difference (%)**	**2016-2017 (n = 2,381)**	**2018-2020 (n = 1,545)**	**Difference (%)**	**Difference-in-difference estimate, adjusted differences, percentage points (95% CI)**
Mood Disorder Diagnosis[a]	380 (7.9)	541 (11.4)	3.5	47 (2.0)	47 (3.0)	1.0	2.4 (-0.1, 4.9)
Mood Disorder Treatment[b]	130 (34.2)	184 (34.0)	-0.2	0 (0.0)	0 (0.0)	0.0	0.0 (0.0, 0.0)

Abbreviations: CI *confidence interval*

Notes: difference-in-difference estimates adjust for maternal age

[a]Pre-test of parallel trends, p = 0.94

[b]Pre-test of parallel trends, p = 0.77

© 2024 Rodriguez MI et al. *JAMA Network Open.*

eTable 8. Changes in Mood Disorder Diagnosis and Treatment Among the Emergency Medicaid Population Following Expansion of Postpartum Care Among Latina Individuals Only, 2016-2020

**Primary Outcomes**	**Women, No. (%)**						
	**Treatment (Oregon)**			**Comparison (South Carolina)**			**Difference-in-difference estimate, adjusted differences, percentage points (95% CI)**
	**Pre-policy (n = 4,815)**	**Post-policy (n = 4,765)**	**Difference (%)**	**2016-2017 (n = 2,381)**	**2018-2020 (n = 1,545)**	**Difference (%)**	
Mood Disorder Diagnosis[a]	380 (7.9)	541 (11.4)	3.5	47 (2.0)	47 (3.0)	1.0	2.4 (-0.1, 4.9)
Mood Disorder Treatment[b]	130 (34.2)	184 (34.0)	-0.2	0 (0.0)	0 (0.0)	0.0	0.0 (0.0, 0.0)

Abbreviations: CI *confidence interval*

Notes: difference-in-difference estimates adjust for maternal age

[a]Pre-test of parallel trends, p = 0.94

[b]Pre-test of parallel trends, p = 0.77

© 2024 Rodriguez MI et al. *JAMA Network Open.*
