# Supplementary material for: Diagnosis of Perinatal Mental Health Conditions Following Medicaid Expansion to Include Low-Income Immigrants
Source: JAMA Netw Open. 2024 Feb 20;7(2):e240062. doi: 10.1001/jamanetworkopen.2024.0062 (PMC10879944; doi:10.1001/jamanetworkopen.2024.0062)
Supplement: Supplement 2. — Data Sharing Statement [file jamanetwopen-e240062-s002.pdf]

Data Sharing Statement

Rodriguez. Diagnosis of Perinatal Mental Health Conditions Following Medicaid Expansion to Include Low-Income Immigrants. *JAMA Netw Open*. Published February 20, 2024. doi:10.1001/jamanetworkopen.2024.0062

Data
Data available: No

Additional Information
Explanation for why data not available: It is not allowed under our DUA
